# The challenge of teaching K-12 genetic principles: A new approach emphasizing polygenic traits, gene-environment interactions, and genetic non-essentialism to improve genetics literacy and reduce racial stereotyping

**DOI:** 10.1016/j.ydbio.2025.09.020

**Published:** 2025-09-26

**Authors:** Jamie R. Shuda, Valerie G. Butler, Robert Vary, Darby W. Sweeney, Fernando L. Wagner, Steven A. Farber

**Affiliations:** a Perelman School of Medicine, University of Pennsylvania, Philadelphia, PA, United States; b Department of Biology, Johns Hopkins University, Baltimore, MD, United States

**Keywords:** Outreach, Education, Teaching, Genetics, Science literacy, Community engagement, Social change, Humane genetics literacy

## Abstract

BioEYES is a K-12 life science outreach program that uses zebrafish to teach Mendelian genetics through hands- on activities. The program has operated for 20+ years, promoting academic equity and fostering scientific literacy for all students. While middle school participants show knowledge gains and improved attitudes about science, ~50% struggle to master foundational genetic concepts. To address this, the authors compared virtual vs. in-person programming, finding minimal differences in learning outcomes but higher gains on simpler survey questions. Rigorous assessments, however, reveal that many students retain only a basic understanding of genetics. Teaching single-gene inheritance, moreover, may reinforce racial stereotypes through “biological essentialism.” To counter this, BioEYES staff will be piloting approaches that focus on human polygenic traits, such as skin color, that is taught alongside a hands-on experiment with a zebrafish pigment mutant. Future efforts emphasize developing curriculum around polygenic traits, gene-environment interactions, and challenging stereotypes about race, which research suggests could improve genetics comprehension while reducing racial stereotyping. We invite community feedback in these efforts to enhance K-12 genetics education strategies.

## 1. Introduction

BioEYES is a K-12 science outreach and teacher education program that uses live zebrafish to teach basic science, genetics, and developmental biology principles. The program prioritizes teachers from low-income schools. Since its launch 22 years ago, over 200,000 students in the U.S., Australia, and China have participated. The program’s success in engaging underrepresented students in science has earned it multiple science education awards from the American Society for Cell Biology (2020), New England Biolabs Passion for Science Award (2019), Genetics Society of America (2018), and the Society for Developmental Biology (2012)

BioEYES’s genetics curriculum is popular among students, educators, and education administrators because students can visualize how traits are passed on from zebrafish parents to their offspring. A cornerstone of the program is that students are participating in an authentic hands-on experience rather than simply learning from a book or a web page. For example, students mate wildtype, heterozygous, and homozygous animals for a Mendelian recessive pigment mutant that lacks melanophores (*nacre*) (Lister et al., 1999), analyze progeny pigmentation, and infer parental genotypes. It is appropriate to highlight the work of the Cheng Lab (2005) in illustrating the power of the zebrafish system to advance society’s understanding of one of the genes responsible for human skin color. Lamason et al. identified the SLC24A5 gene as the causative mutation that underlies the golden zebrafish allele. SLC24A5 accounts for roughly 25–38% of human skin color variation between Europeans and Africans (Lamason et al., 2005). The BioEYES pigmentation experiment demonstrates basic Mendelian inheritance and simplifies complex genetic concepts while still providing rigorous science instruction. It also is a perfect opportunity to discuss the polygenic nature of human phenotypes. BioEYES uses a developmental biology experiment to demonstrate how experimentation, observation, and data gathering leads to scientific conclusions. We contend that this fundamental understanding of the research process helps create a scientifically literate population.

However, starting with single-gene inheritance can unintentionally reinforce racial stereotypes unless paired with concepts like polygenic inheritance and population genetics (Donovan, 2016; Donovan et al., 2020). Can the science community broaden zebrafish genetics education by emphasizing that there is more genetic variation within populations than between them? Emerging research shows that using a “Humane Genetics Literacy” framework can reduce racial prejudices by situating genetics within an economic, social, and political context (Donovan et al., 2024).

Studies show that 7th–12th grade students understood genetics significantly better when taught polygenic models of inheritance and population thinking (populations are not genetic “types” but collections of people that still exhibit genetic variation) compared to the traditional way of teaching Mendelian genetics (Donovan et al., 2020). Others have urged educators to emphasize the sociopolitical dimensions of genetics (Donovan et al., 2024; National Academies of Sciences, 2023). Taken together, to be most effective educators need to teach the basics of genetic inheritance alongside how the environment interacts with an organism’s genes, and the damaging impact of sociopolitical inequalities on marginalized groups. This strategy requires a systemic approach between the formal K-12 education community and STEM outreach partners like BioEYES and includes changes to curriculum, teacher preparation, and a willingness to teach genetics in a new way.

### 1.1. BioEYES’s history

In 2002, BioEYES was founded in Philadelphia in partnership with Thomas Jefferson University and the University of Pennsylvania and now collaborates with multiple large cities, including Baltimore and New York City (Shuda et al., 2024). Each BioEYES site exists in close partnership with an academic institution that has an active zebrafish research community. Demographic data from 2024 indicates that across Philadelphia, Baltimore, and New York City public schools, the majority of students are Black/African American (50%, 71%, and 19.5%), or Hispanic/Latino (24%, 19%, and 42.2%), respectively (School District of Philadelphia, 2024a; Baltimore City Public Schools, 2024; New York City Department of Education, 2024). Across these three cities, BioEYES works with over 6000 students and their teachers each year. The program’s reach is focused on schools that serve underserved students and neighborhoods, but the participating teachers do not often ethnically reflect the students being taught. For example, of the 9349 Philadelphia school district teachers and staff, 58.8% are white, 21.8% are Black, and 3.7% are Hispanic (School District of Philadelphia, 2024b). Given that these young scientists are students from marginalized ethnicities and economic locations, but many of their teachers are not, offers an opportunity to use the BioEYES experience to introduce appropriate discourse around science and race.

Yet despite the success of BioEYES at interesting students in science, increasing their understanding of how to be a scientist, and improving their scientific knowledge, we find that students still have trouble learning genetics. Here we wanted to assess how students that participated in BioEYES remotely during the COVID-19 lockdown compared to students that received an in-person experience. Would there be a link between students’ demonstrated understanding of genetics depending on how they engage with BioEYES? We also wanted to better understand how to improve their comprehension of the subject by redesigning some of the lessons on how traits are inherited, emphasizing that both human and zebrafish skin color are the result of interactions of multiple genes, and exemplifying that skin color is not as simple as black and white. For example, BioEYES teams propose that students explore complex genetic and phenotypic variations within populations followed by an investigation of vertebrate skin color and learning to discuss how a zebrafish genetics experiment is relevant to students’ own inheritance of skin color while distinguishing these genetic principles from the social construct of race. Specifically, if the BioEYES genetics curriculum directly connects polygenic inheritance in the zebrafish to skin pigmentation variations in humans and gene-environment interactions while refuting assumptions about racial differences, will it increase students’ comprehension of genetics?

## 2. Results

### 2.1. Student learning goals and outcomes for virtual BioEYES

Before diving deeper into race as a social construct, it is important to understand that in addition to BioEYES’s mission of having students become scientists through hands-on experimentation, one of the learning goals is to introduce the molecular pathways of genes and how they lead to different phenotypes and genotypes, using skin pigment inheritance as an observable trait. Moreover, the COVID-19 pandemic created a challenge for us to deliver BioEYES programming and to maintain teacher engagement. In response, BioEYES program staff adapted its in-person genetics curriculum for middle school, described as the “Intermediate” level, to a virtual platform. The team developed three virtual modules in Google Slides, and utilized educational tools like NearPod, PearDeck, and Jamboard to increase student interactivity and engagement. Staff piloted the units in spring 2020 and solicited feedback that year from classroom teachers in spring and summer. Then after integrating the feedback, BioEYES fully implemented the modules beginning in the 2020–2021 school year. The content for each module is outlined in Table 1.

**Fig. 1.**
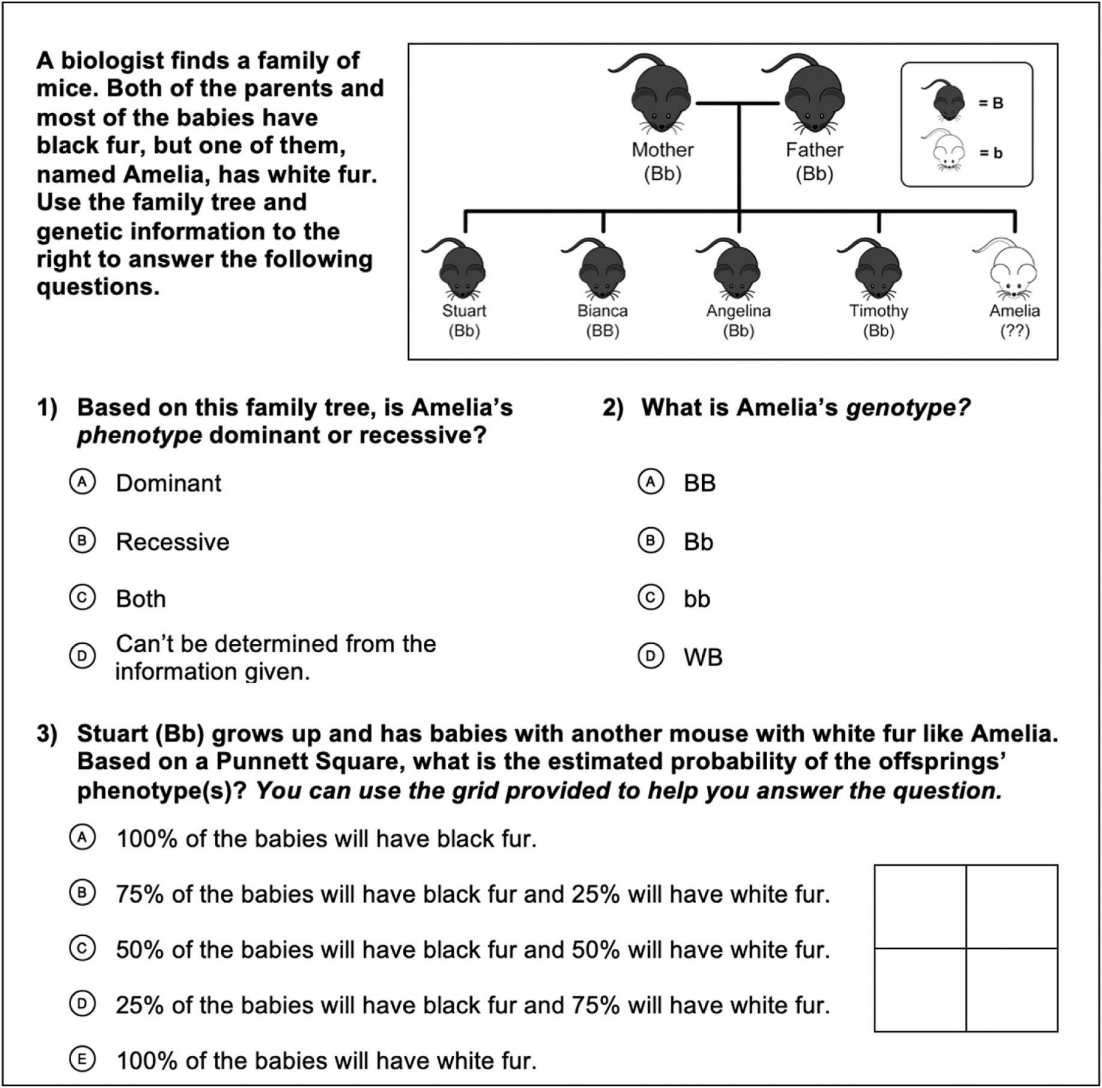
Intermediate (7th/8th grade) student assessment questions, 2019–2023.

To assess the effectiveness of BioEYES genetics on student learning and attitudes about science, BioEYES asked the same questions before and immediately after programming. Over time the questions asked of students to assess genetics knowledge (Fig. 1) have varied. This approach allowed the team to determine the students’ baseline knowledge and measure the impact of the BioEYES experience on their learning. Staff also compared knowledge gains in prior years of in-person BioEYES to those observed in the virtual modules. Upon comparing in-person and virtual results, we observed significant differences between the two delivery methods (Table 2).

Significance was estimated for all questions using a two-tailed, paired *t*-test and was FWER-corrected using the Bonferroni correction. The comparative difference shows the change in effect size between in-person and virtual students, analyzed for significance using a two-tailed, unpaired *t*-test.

In this analysis of the genetics assessment data, the first question, I-K1.1 (also noted below for clarity), showed a significant positive change for the in-person program, but not for the virtual program. The second question, I-K2.1, showed a significant positive change in both versions. The third question, I-K3.1, did not show a significant change in either version. Only I-K1.1 showed a significant difference in the level of improvement between the in-person and virtual programs, with a p-value of 0.049. The most recent iterations of these questions (discussed below) switched the animal from zebrafish pigmentation to mouse coat color to test the degree to which students could transfer what they learned from zebrafish to other animals. This is the most rigorous test to gauge if students deeply understand the genetic concepts.

- I-K1.1: *Based on this family tree, is Amelia’s phenotype dominant or recessive?*
- I-K2.1: *What is Amelia’s genotype?*
- I-K3.1: *Stuart grows up and has babies with another mouse with white fur like Amelia. What is the estimated probability of the offsprings’ phenotype (s)?*

One notable difference between the two versions is that the virtual students scored better on the PRE assessments on the first two questions than did in-person students, meaning that there was less room for improvement. For instance, question I-K1.1 had the in-person students improve by a significant 9.9%, while the virtual students improved by an insignificant 4.5%. However, note that the in-person students started with 67.7% correct answers on the PRE, while the virtual students started with 84.0%. Even though the in-person students showed more change, the virtual students still had higher POST scores (77.5% in-person vs. 88.4% virtual).

Regardless of this outcome on the first two questions, the third question failed to get significantly positive results from either version of the program, and in contrast to the previous two questions, the two cohorts of students got nearly identical results on the PRE assessments. To further explore this, the team analyzed the various “Punnett square” questions that were asked in the Intermediate student assessments over the past 15 years.

### 2.2. Analysis: 15 Years of BioEYES’s Punnett square data

The BioEYES pre and post student assessments have consistently shown knowledge gains across all grade levels and subjects for over a decade (Shuda et al., 2016). The questions have often been updated or modified to improve clarity, better reflect the subjects being taught, and accommodate program changes, among other reasons. The most notable gains have been on questions that asked the students to define a term (e. g., “An organism that inherits two copies of the same allele is considered:” *Answer: Homozygous*), or to retrieve a concrete piece of information (e.g., “How many chambers does a HUMAN heart have?” *Answer: Four*). As expected, it has been more challenging to demonstrate significant changes in the students’ understanding of the underlying genetic processes and concepts.

For instance, since 2009, BioEYES has asked the Intermediate students five different versions of a question to probe their knowledge of inheritance and Punnett squares. This extensive analysis is contained in Appendix A. In short, while statistically significant gains emerged following BioEYES genetics, as the questions got harder (higher degree of abstraction), staff observed smaller gains when probing for conceptual understanding. Having settled on the conceptual questions, the BioEYES teams around the world want to explore ways to increase genetics knowledge gains without promoting an over simplistic view of genetic inheritance (monogenic inheritance) that can promote racial stereotyping. Is such an approach even possible in a week of programming? Should BioEYES staff modify the way they teach the program? Are there any clues about how to make such changes from the education literature?

## 3. Discussion

### 3.1. In person vs. virtual instruction

After teaching middle school genetics for over 20 years and publishing BioEYES’s student outcomes, we have sought to understand why half of participating students continue to have difficulty with the subject, and strategies we could implement to improve their genetics mastery. First, we compared the in-person and virtual instructional models.

While we saw greater knowledge gains with the in-person modules, it is unclear why the virtual students tended to have higher initial scores than in-person students on questions I-K1.1 and I-K2.1 (Table 2). Interestingly, the virtual modules were delivered in three days, while the in-person unit spanned five days (three with a BioEYES Educator, and two with the classroom teacher). During the COVID-19 pandemic, computer and internet access were not readily available for many students. It’s possible that those who had access had the advantage of more resources from the internet or others in the home compared to the in-person students. Or, perhaps, teachers were more likely to use the virtual programs as a review of genetics, while the in-person programs served as an introduction to the subject. Thus, some students may have had pre-existing knowledge of genetics, or their teacher may have included additional genetics content during the BioEYES week, both of which could have affected the survey results. Alternatively, it’s possible that less-motivated or less-engaged students who participated in the in-person programs were not present during the virtual modules. Many of the teachers noted that they frequently had half or fewer of their scheduled students present for virtual classes, which may have resulted in the virtual cohort consisting mainly of highly engaged students who were more likely to score higher. Also, a subset of virtual students may have taken part in BioEYES in prior years more so than the in-person students and their pre-existing knowledge, understanding, and comfort-level in working with zebrafish may have helped boost the initial scores of the virtual cohort. While the grade level units do vary, the scientific techniques and background information about zebrafish are similar.

### 3.2. How do students experience genetics?

BioEYES’s data consistently shows that middle school students demonstrate content knowledge gains about zebrafish genetics after participating in BioEYES. However, as illustrated by the data (Appendix A) it also reveals that about half of the students still struggle to apply what they have learned about zebrafish pigmentation genetics to mouse coat color genetics (something not discussed during the BioEYES experience). We consider this a “high bar” of conceptual understanding. While students learn definitions and can correctly complete Punnett squares, they find it much more difficult to demonstrate their understanding of the processes involved, or to apply their knowledge to a new context, such as a family of mice.

Many researchers have noted that genetics is an extremely challenging subject for students of all ages (Duncan and Reiser, 2007; Kindfield, 1992; Lewis and Kattmann, 2004; Lewis et al., 2000b; Marbach-Ad and Stavy, 2000; Stewart and Van Kirk, 1990; Tsui and Treagust, 2003; Venville and Treagust, 1998). Middle and high school students often harbor misconceptions about genetics (Lewis and Kattmann, 2004; Lewis and Wood-Robinson, 2000; Stewart, 1982; Williams et al., 2011). For example, high school students frequently struggle to explain basic dominant and recessive patterns of inheritance (Mills Shaw et al., 2008), often fail to consider the cellular or molecular mechanisms underlying inheritance patterns (Castro-et al., 2022), and conflate genes, proteins, and chromosomes (Lewis et al., 2000a).

While children as young as seven years old can understand kinship and that parents and their children share similar traits (Solomon et al., 1996), middle school students, like high school students, often do not grasp the genetic mechanisms that underlie these similarities or how genes transfer information. This can lead to the simplistic notion that equates genetic material with a visible trait and that the trait itself is what gets passed on to the next generation. Additionally, middle school students tend to focus on one allele’s role in producing a phenotype, without considering the contribution of the second allele (Duncan et al., 2016).

Researchers have also found that middle school students misunderstand the relatively equal genetic contributions of parents to their offspring (Williams et al., 2011). Many students in the study thought that girls received their traits from their mother and that boys inherited their traits from their fathers. They also believed that mothers contribute more to her offspring than fathers do. It is anticipated that having direct instruction and conversations around how inheritance of skin color is also found in all of us may help formalize this scientific principle for the students. Researchers suggest that educators should focus on simplified examples of genes and proteins at the cellular level in middle school to prepare students for more advanced instruction on these concepts in high school (Castro-et al., 2022; Duncan et al., 2011). The team wondered if perhaps the abstract nature of genetics was a barrier to student learning. However, research shows that students are capable of reasoning about abstract concepts such as genetics, even at the middle school level (Castro-et al., 2022; Duncan et al., 2011; National Research Council [NRC], 2007; Venville and Donovan, 2007). This gives us confidence that students can use the BioEYES scientific experience to bridge what their zebrafish larvae’s phenotype is and how it relates to their own skin color. This may be the first time for many children that they see their own ethnicity through a scientific, not just a socially constructed, lens.

### 3.3. More genetics education is needed across K-12

The authors argue that a greater “dosage” of genetics may be required over multiple grades. Students should be given sufficient opportunities to explore the subject in a scaffolded manner, starting in elementary school and continuing through high school. To help students develop a mastery of genetics, researchers have proposed learning progressions (LPs) for grades K-12 (Roseman et al., 2006; Duncan et al., 2009; Elmesky, 2013) and refined for grades 4–12 (Duncan et al., 2017; Castro-et al., 2021; Todd and Kenyon, 2016). These LPs are designed to give repeated opportunities for students to wrestle with genetics concepts and provide a coherent sequence of genetics instruction.

However, some questions about how to teach genetics have yet to be settled. The four current genetics education models (Duncan et al., 2009) are:

1. The meiotic model describes the physical mechanics of inheriting DNA from one’s parents
2. The molecular model illustrates the relationships between genes, proteins, and chromosomes, and how altering a protein results in a phenotype
3. The inheritance model (Mendel) is based on readily observable monogenic phenotypes
4. The modern complex polygenic model which includes how the environment interacts with multiple genes to produce specific traits

Some scholars have proposed that the meiotic and molecular models should be taught before high school, where they are typically first introduced (Duncan et al., 2016; Roseman et al., 2006). However, there remain many questions as to which grade level and model to deploy. Middle school students have stronger learning gains when genetics is introduced using the molecular model (Roseman et al., 2006). Evidence from Duncan, Castro-Faix, & Choi (Duncan et al., 2016) suggest that molecular genetics should be taught before Mendelian genetics in middle school, as students in their study were able to use their knowledge of molecular genetics to understand Mendelian inheritance. The results were weaker when Mendelian genetics was taught prior to molecular genetics. In high school, teaching the molecular model first has been associated with modestly higher gains in overall understanding than those taught with the classical model (Mendelian + Meiotic) first, although the differences were not significant (Duncan et al., 2017). Another study found that high school students who learned the genetic model of simple dominance followed by the meiotic model had a better understanding of the meiotic model and were able to use it to solve complex genetic problems involving atypical data (Wynne et al., 2001). However, it remains unclear in what order the genetics, meiotic, or molecular models should be taught, and at what grade level(s) (Duncan et al., 2016; Duncan et al., 2009; Duncan et al., 2017; Castro-et al., 2021)?

While BioEYES has historically taught Mendelian genetics (inheritance model) because it is thought to be easier for young learners to understand, the emphasis that one gene explains a complex trait does not accurately reflect how multiple genes work together to drive most phenotypes, which is certainly the case for skin color (Dougherty, 2010; Kampourakis, 2017; Jamieson et al., 2013; Redfield, 2012).

Given the difficulty of learning genetics, could student comprehension be improved by introducing a framework most compatible with social change (modern complex model) to how the BioEYES teams are currently teaching genetics? That is, by teaching the complex polygenic model of genetics first, teaching about genetic variability within “races,” and then speaking directly to students about their own melanin inheritance and giving them the knowledge and tools to investigate how skin color manifests in zebrafish, would they understand genetics on a deeper level? What other strategies might BioEYES staff adopt?

### 3.4. Improving genetics literacy

BioEYES staff introduced the molecular model to the middle school students in the 2022–2023 school year with the hope of deepening their understanding of inheritance. For example, two pages were added to BioEYES’s middle school student journals to include text and diagrams explaining where genes are located, and how they work. The content covers cells, DNA, genes, and chromosomes on one page, and transcription, translation, gene expression, proteins, traits, and phenotype on another page. In the 2022–23 school year, the BioEYES educators also made slight changes to their instruction to better integrate these concepts. They had students read the new content aloud or independently and used the term “representation” when referring to the letters (e.g., Aa, AA, aa) that stand in for the alleles. However, due to time constraints, it was primarily up to the classroom teacher to review the new journal content with the students, to answer questions, and correct misconceptions. BioEYES educators balance their time in the classroom between instruction and hands-on activities that the students perform (e. g., collecting embryos, looking under the microscopes, caring for the embryos) because they have found the latter to be crucial for sparking students’ engagement in science (Shuda et al., 2016).

Additionally, students were not expected to memorize or to fully know all the new content. The goals were: To help students begin to understand the molecular basis of genes and how they give rise to observable traits like pigmentation, to introduce molecular concepts so they have some familiarity with the components by the time they enter high school, and to provide an experience where the students are actively engaged as scientists exploring issues that impact their own lives.

### 3.5. Future proposed changes to BioEYES

The authors propose the following curricular and instructional changes in future phases to improve students’ genetics understanding:

- **Polygenic nature of most traits:** To better reflect the true nature of genetics and convey that more than one gene is typically involved in producing a trait, the team recently developed several resources that best align with the modern “complex model” of genetics education. We have included a presentation (Appendix B), a simulation game where students “make” a smoothie (Appendix C), and a lesson plan (Appendix D). These resources can be shared with teachers at professional development workshops and with students in the classroom. They can be utilized in combination or separately with BioEYES activities.

**Fig. 2.**
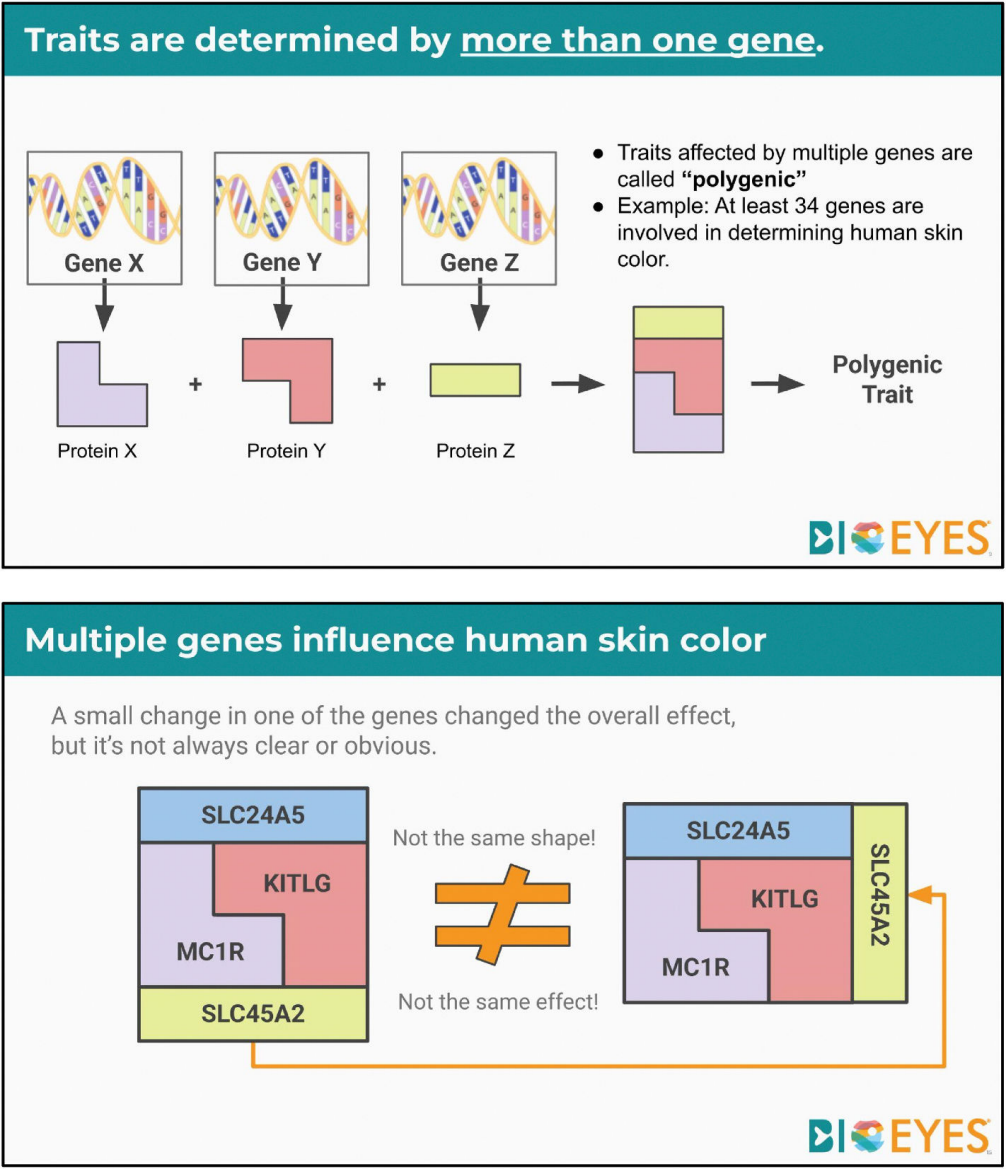
Instructional slides from Appendix B. (A) A diagram of a fictitious polygenic trait. (B) A diagram of four genes identified in human skin color variation that illustrates how gene changes can produce different effects.

The presentation (Fig. 2 and Appendix B) explores the molecular nature of skin color genetics in humans and zebrafish. This is an example of how multiple genes can combine to create variations in phenotypes. It also gives students time to discuss how polygenic traits might challenge ideas of fixed racial groups.

The Smoothie game (Appendix C) utilizes a smoothie to illustrate how multiple genes interact to produce a specific phenotype. For example, different ingredients (representing the individual genes) can be combined in different quantities (alleles) and blended (gene expression) to produce a unique smoothie (phenotypes). Despite the variation, the outcome is the same: It’s still a smoothie. This game provides educators with a fun framework to discuss molecular genetics with students and how multiple genes can interact to create a complex phenotype such as skin color.

The objective of the lesson plan (Appendix D) is for students to explain how phenotypic variation is a result of complex interactions between multiple genes and the environment, with the scientific question being: “How do our bodies know what color our skin should be?”

- **Gene-environment interactions:** Convey an understanding to students that the environment affects genetic traits and thus, genes are malleable. For the smoothie game described above, environmental factors that produce a variation of phenotypes can be discussed such as fruit ripeness, temperature, and whether you blend or puree a smoothie.

A discussion could also be had about how the environment historically affected skin color through the selection of specific beneficial alleles. E.g., people that lived closer to the equator where the sun is brighter, and how they had more melanin. But as populations moved elsewhere, genetic mutations followed by selection resulted in lighter skin color so more Vitamin D could be produced from the sun’s ultraviolet rays.

- **Teacher workshop changes:** Keep the hands-on exploration with zebrafish (catching fish, collecting embryos, making observations, and collecting data) but modify the teacher professional development workshops to incorporate the new content described above with the teacher guidelines the authors have created (Appendix E). The guidelines include: 1) The nature and history of genetics, 2) that Mendelian genetics (one gene equals one trait) is not applicable for many readily observable phenotypes that often result from multiple genes (polygenic; e.g., height, skin color, …), 3) gene-environment interactions, and 4) how teaching Humane Genetics Literacy (described below) can reduce stereotypes about skin color (Appendix E).

Brian Donovan developed the Humane Genetics Literacy model (Donovan et al., 2024) which builds upon the use of polygenic and gene-environment interactions, but adds an explicit emphasis that more genetic diversity occurs within races than between them. Evidence from this emerging research has shown that this model can minimize genetic essentialism. It would be extremely challenging (and maybe impossible) to conduct an evaluation of teachers (and students) to assess if their stereotypes and misconceptions about race change because of these revisions to BioEYES instruction.

- **Misconceptions:** BioEYES staff could also work with teachers to create lessons that highlight and refute misconceptions about genetics or race. Lewandowsky et al. (2012) provide a useful strategy for doing so: 1) Give students a warning that the (mis)information you are about to present is not true, provide a correction that is repeated sufficiently in order to strengthen it in students’ minds, and provide evidence for why the information is inaccurate (Lewandowsky et al., 2012). In the coming year, the BioEYES team will develop an assessment that explores the degree to which our programing can impact common genetic misconceptions.
- **Punnett squares:** BioEYES might develop short lessons on Punnett squares that aids students to conceptualize what the letters represent and consider the process by which two alleles give rise to traits. While another lesson could review the math skills needed to interpret Punnett square results, followed by practice exercises.
- **Seek feedback:** Recruit education stakeholders in varying roles (i.e., teachers, partners, experts, researchers) in the design and development phases to offer feedback. Ideally, stakeholders will reflect the demographics of participating students to provide perspectives that may not be considered by others. This will offer insight into the best practices for teaching about skin color that nurtures and supports the identities of the students being served.

By offering additional workshop content, the team can ensure that teachers and administrators are comfortable with the new changes to BioEYES Genetics while also providing the reasoning behind them: For the purpose of improving students’ understanding of the complexity of genetics. Rigorous assessments will need to be developed to understand the effect of these efforts.

#### Polygenic vs. single gene mendelian inheritance:

To clarify, skin color is a *polygenic* phenotype, while “race” is influenced by skin color but is a social construct (Donovan et al., 2024). In this context, modern multifactorial polygenic inheritance defines complex traits as resulting from many factors that can be influenced by interactions between genes and environments that include socioeconomic (e.g., quality of neighborhood, income), political (e.g., access to resources and healthcare), and environmental inputs (e.g., diet, microbiota) (Mackay et al., 2009). Cardiovascular disease is one example of a multifactorial genetic disease in that the disease severity is impacted by specific gene variants (e.g. LDL receptor alleles), but also environmental factors such as smoking, exercise, and nutrition. One could argue that it is far more difficult to maintain a healthy lifestyle when working three jobs and not having access to or being able to afford healthy food or a gym membership.

Several scholars have called for reforms in genetics education arguing that an exclusive focus on simple single gene Mendelian genetics, while a fundamental principle, fails to explain common phenotypic variation in the human population. Instead, it is better to emphasize that most traits have polygenic origins and are often influenced by the environment (Dougherty, 2010; Kampourakis, 2017; Jamieson et al., 2013; Redfield, 2012). Another strategy is to also train teachers on new ways to reduce genetic essentialism while simultaneously teaching complex genetics (Appendix D & E). Providing an opportunity for teachers and students to examine how social and environmental factors influence our genetics (e.g., how segregation affects health outcomes) makes it much more difficult for individuals to claim that genetic differences between races exceed those within them, a belief that fuels racial stereotypes (Donovan, 2016; Donovan et al., 2020, 2024).

### 3.6. How can BioEYES be taught to empower ALL students to become change agents regardless of their background?

While BioEYES has always been focused on equity and access, its well-meaning genetics curriculum with its centeredness on single gene Mendelian inheritance, may be unintentionally inhibiting students’ genetics literacy and amplifying genetic essentialism and racial biases. Thus, the authors will be piloting and testing the new approaches outlined in section 3.5.

The primary goal of the program is for students to be actively engaged in their learning so they can envision themselves as scientists, rather than simply absorbing information from educators and scientists. BioEYES collaborates with teachers and school administrators to create a welcoming environment for everyone, with no recruitment process for students. All students whose teachers partner with the program can participate. Program staff intentionally support economically disadvantaged communities and do not charge them; instead, we use institutional power and privilege for good. BioEYES raises funds to provide rigorous science education, student-centered experiences, and supplies for students and teachers. In addition, staff differentiates the content by creating varying levels of curricula and activities tailored to the abilities of the students. The program follows accessibility guidelines for disabled populations (e.g., in how the student journals are designed, by projecting microscopic images on a wall for the visually impaired, etc.) and offers the content in Spanish. Since COVID-19 and the integration of Zoom and Teams as teaching platforms, staff have expanded the opportunities for volunteers to engage with students and have actively recruited volunteers from different backgrounds to interact with them in-person and virtually.

The very best practices must be employed for the populations being served. One example is staying current with education research and incorporating its recommendations for curricular and instructional reform. And as part of the program’s commitment to serving all students regardless of background or zip code, author V. B. published an article in *The Node* about how to become a culturally relevant science educator (Butler, 2017). Such an educator endeavors to truly learn about the students they are teaching, which can be challenging when an outreach educator is in the classroom for a limited time. They model a nurturing attitude and allow students to share their ideas openly while celebrating their cultures and communities. They also seek out ways to incorporate the unique backgrounds, ancestry and experience, while maintaining high learning expectations. These practices build strong identities, a sense of belonging, new perspectives, critical thinking, greater engagement, and ultimately, stronger learning gains for students.

### 3.7. Potential challenges

It will be important for BioEYES staff to conduct extensive planning, testing, and evaluation as it implements the modifications described in this article. Not doing so may create negative effects and challenges, such as:

- If the classroom teacher has not taken the time to adequately get to know their students as individuals and to develop relationships with them, the students may not want to engage in discussions that challenge their ideas and beliefs. This disconnect deters critical thinking.
- Talking about skin color and race directly is not easy, and especially if stakeholders are not ready for it, and if not done with sensitivity and based on best practices, evidence-based research, and expert advice. The job of BioEYES educators will be to facilitate civil discourse and exposure to different perspectives. Because racism causes discomfort for students of color, race should be addressed directly *in a supportive environment*.
- Amplifying racial stereotypes and genetic essentialism, especially if individuals have strong beliefs in these areas to begin with. If a person’s worldview is challenged, it can sometimes backfire and result in reinforcing the individual’s belief or misinformed idea.
- If the classroom teacher does not have time to do the activities outside of BioEYES Genetics, students may have less of an opportunity to improve their genetics comprehension and knowledge.
- Pushback from stakeholders and administrators for teaching genetics differently. If a clear rationale is not elucidated, administrators may not understand why the authors seek to underemphasize single gene inheritance in favor of polygenic inheritance.

Is it even possible to incorporate these adaptations into a one-week program? Or are such reforms too ambitious for BioEYES? By not changing how the scientific community teaches genetics, students will continue to have difficulty understanding it. As Frederick Douglass said, “If there is no struggle, there is no progress” (Douglass, 1857).

Internal discussions about growing BioEYES have always focused on the balance between reaching more students and expanding the content to create deeper learning experiences. Given BioEYES’s success at sparking student engagement in science, staff have prioritized reaching more students. However, the challenge of teaching K-12 genetics, along with the need for multiple exposures over several school years, means this tension remains.

The proposed changes discussed throughout this paper may increase students’ understanding of genetics and refocus staff’s teaching time to connect the dots for students that skin color is a direct result of biological processes called genetic inheritance, and that there are many factors that contribute to one’s genetic makeup. The authors have identified evidence-based strategies to implement that may counter racial stereotypes. The team currently sees positive impacts on students’ attitudes toward science and science careers but are curious if these changes can help students build confidence in science and an understanding of genetics. Will they be more invested in understanding inheritance if they see how it applies to them?

Over the past twenty years BioEYES has leveled the playing field for all students to become scientists in their own classroom. Students learn how to engage with research and conduct experiments that explore the inheritance of black pigment through a scientific lens. This experience exemplifies how race is a social construct, but skin color is a genetic phenomenon largely determined by *a collection* of specific gene variants. Empowering all students to understand the interconnectedness between genes, inheritance, skin color, the environment, and emphasizing the genetic similarities among ethnic groups can spark conversations around social change. However, program staff need to continue to monitor the program’s student outcomes and work to improve students’ overall comprehension of genetics, while being mindful of reducing racial biases.

We invite the community to provide input on ways science educators might help students succeed in genetics. To comment, please see the community page at www.bioeyes.org.

## 4. Materials and methods

### 4.1. Zebrafish husbandry

#### University of Pennsylvania:

The zebrafish procedures were approved by the University of Pennsylvania Animal Care and Use Committee (Protocol #804450). Zebrafish stocks of wild-type AB and *nacre* pigment mutants were maintained using a modern recirculating aquarium facility. Student data collection has been approved by the University of Pennsylvania’s Institution Review Board (Protocol #843873). The School District of Philadelphia (SDP), through the Office of Research and Evaluation’s (ORE) Research Review Committee, has approved study #2022–10-1050, “Project BioEYES & DrosoPHILA: The Impact of Science Outreach on K-12 Students.”

#### Fontbonne University:

All Zebrafish (Danio rerio) protocols were approved by the Washington University Animal Care and Use Committee (Protocol #22–0076 “Developmental Biology Educational Outreach to St. Louis Public Schools”) where the zebrafish are housed in St. Louis, MO in the Washington University Zebrafish Facility. Zebrafish stocks of wild-type AB and *casper* pigment mutants were maintained using a modern recirculating aquarium facility.

### 4.2. Virtual modules

**Fig. 3.**
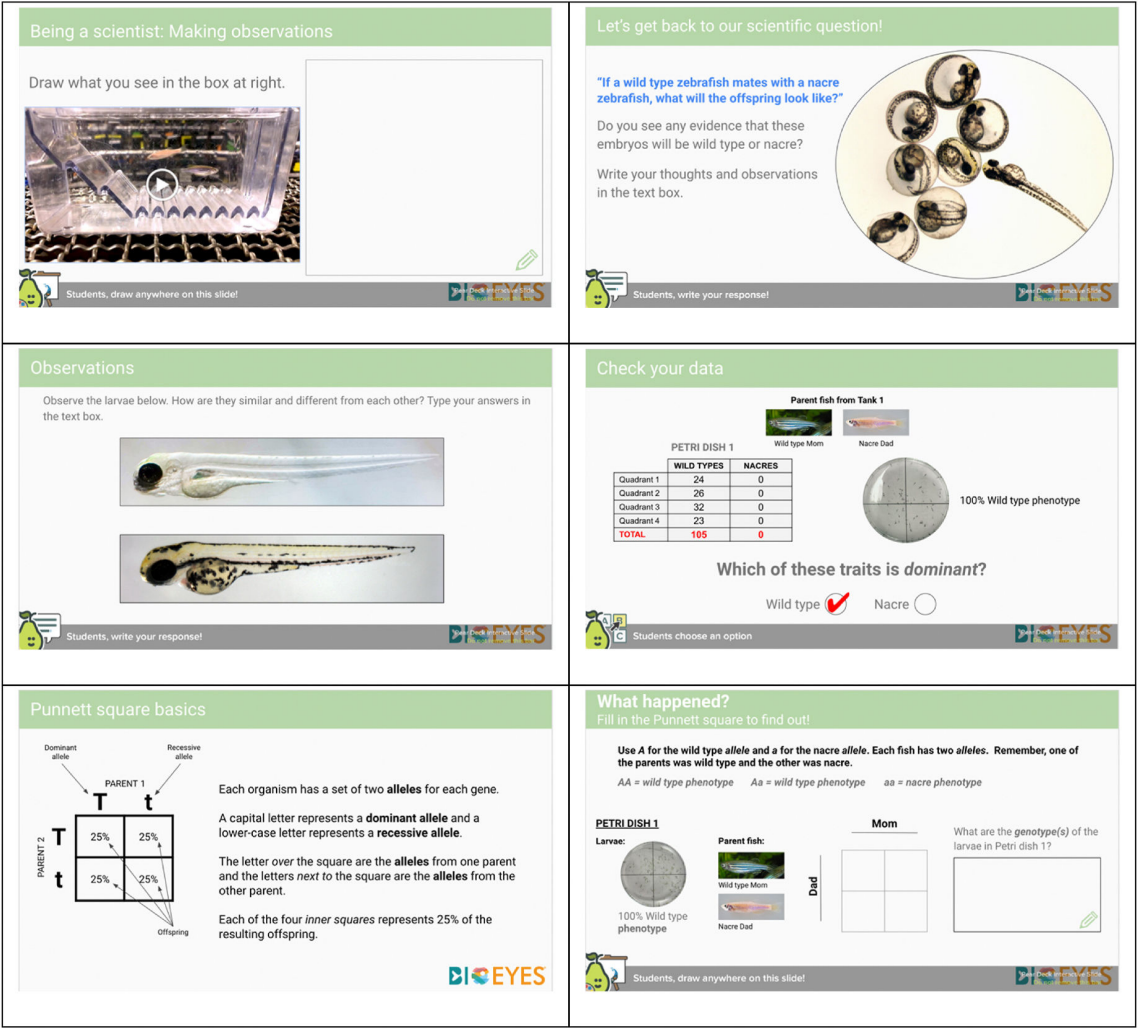
Sample slides from Intermediate (7th/8th grade) Virtual BioEYES modules.

**Fig. 4.**
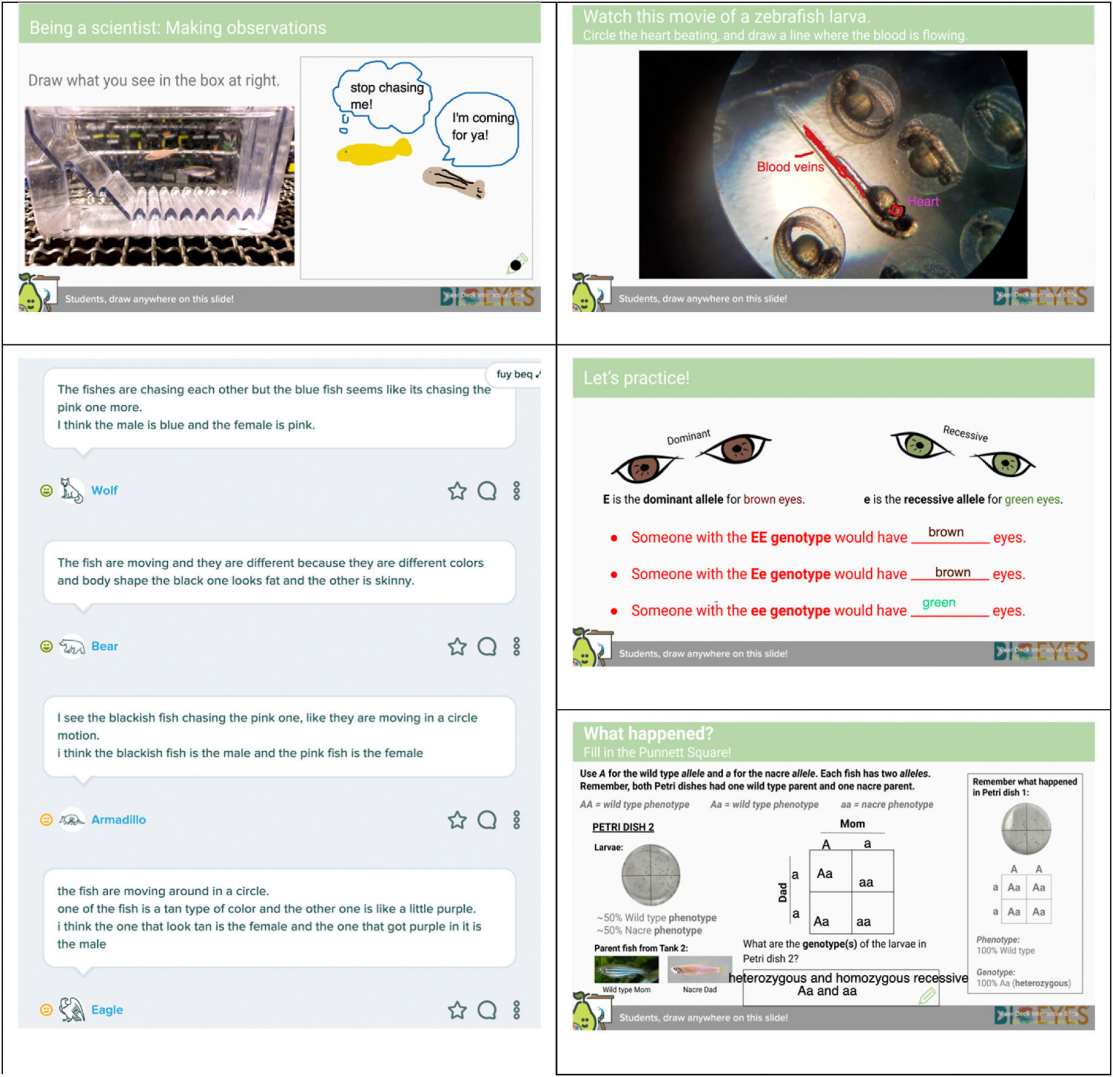
Examples of student work from the Intermediate (7th/8th grade) Virtual BioEYES modules.

The Virtual BioEYES Genetic modules for the middle school level were piloted with a small cohort of students in April 2020 and implemented fully in the 2020–2021 school year. These modules used Google Slides and optional Pear Deck extensions to present an interactive online lesson that the students could complete synchronously (led by a live presenter such as their classroom teacher or a BioEYES Outreach Educator) or asynchronously (self-led, at the students’ own pace). See Figs. 3 and 4 for examples of slides from Virtual BioEYES and resulting student work. The full modules for all levels can be viewed here.

The modules use the interactive Pear Deck extension that allows students to type in responses, make selections, and draw observations that the presenter receives in real time.

The in-person program has existed since 2002 and offers a virtual BioEYES experience was new territory for BioEYES staff and for the collaborating teachers and students. With online programming, we discovered that there was limited or no time for informal conversations with the students. This unstructured time was often an opportunity for students to ask individual questions in person and for the educator to talk with each small group of 3–4 students. For example, during in-person sessions, when students are waiting for their turn at the microscope, the BioEYES educators often check students’ understanding of concepts and answer any questions. This informal teaching time is important in a very busy classroom setting. Also, bringing BioEYES to a classroom means there is a new instructor, and we believe this novelty can capture student interest as well. Thus, engagement and curiosity are capitalized on when the program is delivered in person because there are more opportunities to build relationships with students, a tenet of culturally relevant teaching.

Virtually, student engagement was much harder to achieve and measure in real time. Cameras were turned off (was the student even there?), students experienced computer fatigue and distractions, and many students had to share a computer, if they had one, with a parent or sibling.

### 4.3. Pre and post assessments

To judge the effectiveness of BioEYES’s programming, all students take knowledge and attitude assessments prior to the program (“PRE assessments”) and immediately following the program (“POST assessments”). We wanted to compare the virtual genetics program to the in-person delivery model. Due to the aforementioned limitations of the virtual format, and the educators’ experiences in leading the virtual modules, we hypothesized that the student assessments would show less improvement in content knowledge with the virtual modules compared to those from the in-person program.

To compare the two sets of data, the results were analyzed from the 2019–2020 school year—the last school year that was fully in-person—and compared them to the virtual-only results from 2020 to 2021. As the only assessment data for the virtual program came from the University of Pennsylvania and Fontbonne University BioEYES centers, the in-person data was limited to those two centers as well to control for differences in teaching style or target student bodies between locations.

### 4.4. Statistics

The *n* for each question included only those students who completed both a PRE and a POST assessment with identifying student numbers that could be matched up. The in-person results had many more data points than the virtual results (n = 832 and n = 380, respectively), so a series of three analyses of ~380 randomly selected, in-person data points was performed to make certain that any differences in statistical significance was not the result of differing n values. As the random analyses produced similar statistical results to the full analysis, the complete set of data was used for the results.

Significance is estimated for all questions using a two-tailed, paired *t*-test and FWER-corrected using the Bonferroni correction. The level of improvement for each set of data was calculated by assigning a value of “1” to all correct answers and “0” to all incorrect or blank answers and subtracting the PRE value from the POST for each response. The resulting lists of differences for each question from the in-person and virtual program results were analyzed using a two-tailed, unpaired *t*-test. This allowed us to see if the level of improvement shown by the virtual program was significantly different than that shown by the in-person model.

## Supplementary Material

Appendices A-E. Supplementary data

## Figures and Tables

**Table 1:** Middle School (“Intermediate”) Virtual BioEYES Genetics content.

Module 1: Designing a zebrafish genetics experiment	Module 2: Zebrafish development	Module 3: Practicing Mendelian genetics

• Essential vocabulary	• Essential vocabulary	• Essential vocabulary
• Introduction to scientific inquiry and experiments	• Demonstration of how zebrafish eggs are collected in the lab	• Exploring the anatomy of zebrafish larvae
• Information on zebrafish and why they are used in scientific research	• Counting eggs in a Petri dish	• Viewing the heartbeat and blood flow of larvae
• Explanation of phenotype and genotype (wild type = stripes, nacre = no stripes)	• Learning the “anatomy” of zebrafish embryos	• Introduction to Mendelian genetics
• Setting up an investigation by posing a question and forming a hypothesis	• Exploring the stages of zebrafish embryonic development	• Collecting data regarding the phenotypes of the larvae, including counting the larvae
• Exploring how breeding is set up in the lab	• Observing zebrafish embryos, including the different features	• Applying this data to work out genotypes with Mendelian genetics
• Recording observation of the adult fish; looking at the differences between male and female, wild type and nacre; as well as studying their behavior		• Making a conclusion to the investigation, including reflecting on hypothesis from Module 1.

**Table 2:** Student assessment results for the in-person and virtual Intermediate (7th/8th grade) programs.

Intermediate Knowledge Question		n =	% Correct Pre	% Correct Post	Difference	Adjusted p-value	Comparative difference	Comparative p-value
I-K1.1 Based on this family tree, is Amelia’s phenotype dominant or recessive? (Answer: Recessive)	Virtual	380	83.95%	88.42%	4.47%	0.088	− 5.39%	0.049
	In-person	832	67.67%	77.52 %	9.86%	< 0.001		

I-K2.1 What is Amelia’s genotype? (Answer: bb)	Virtual	380	80.53%	85.79%	5.26%	0.037	− 0.03%	0.993
	In-person	832	71.03%	76.32%	5.29%	0.011		
I-K3.1 Stuart grows up and has babies with another mouse with white fur like Amelia. What is the estimated probability of the offsprings’ phenotype(s)? (Answer: 50 % white, 50 % black)	Virtual	380	48.42%	54.21%	5.79%	0.133	4.47%	0.205
	In-person	832	47.48%	48.80%	1.32%	1.549		

## Data Availability

Data will be made available on request.

## Appendices A-D. Supplementary data

Supplementary data to this article can be found online at https://doi.org/10.1016/j.ydbio.2025.09.020.
