# Supplementary material for: The challenge of teaching K-12 genetic principles: A new approach emphasizing polygenic traits, gene-environment interactions, and genetic non-essentialism to improve genetics literacy and reduce racial stereotyping
Source: Dev Biol. Author manuscript; Available in PMC 2026 Jun 7. (PMC13242738; doi:10.1016/j.ydbio.2025.09.020)
Supplement: Appendices A-E. Supplementary data [file NIHMS2169576-supplement-Appendices_A-E__Supplementary_data.docx]

**Appendix A - Discussion of knowledge of inheritance and Punnett squares learning assessment**

The first four versions (Table Appendix A Table 1A) all had the same four multiple choice answers and the same correct answer **(B)**.

**Appendix A, Table 1. “Punnett Square” question results on Intermediate (7th/8th grade) BioEYES student assessments, 2009-2017.**

| **A** | (A) | *A* | *A* |  | (B) | *A* | *a* |  | (C) | *a* | *a* |  | (D) | *Ab* | *Ab* |  |
| --- | --- | --- | --- | --- | --- | --- | --- | --- | --- | --- | --- | --- | --- | --- | --- | --- |
|  | *A* | AA | AA |  | *A* | AA | Aa |  | *a* | aa | aa |  | *Ab* | AAbb | AAbb |  |
|  | *a* | Aa | Aa |  | *a* | Aa | aa |  | *a* | aa | aa |  | *Ab* | AAbb | AAbb |  |
|  |  | | |  |  | | |  |  | | |  |  | | |  |

| Knowledge Question | | n= | % Correct Pre | % Correct Post | Difference | Percent Change | p-value |
| --- | --- | --- | --- | --- | --- | --- | --- |
| **B** | IK6.0 – If rolling your tongue is a recessive trait, which answer would show a 25% chance of parents passing on the gene? (2009-2010) (Answer: B) | 1826 | 48.0% | 69.8% | 21.7% | 45.2% | <0.001 |
| **C** | IK6.1 – If rolling your tongue is a dominant trait, which answer would show a 3:1 ratio of parents passing on the trait? (2010-2011) (Answer: B) | 1242 | 43.0% | 53.7% | 10.7% | 24.9% | <0.001 |
| **D** | IK6.2 – Which answer shows the inheritance of a recessive trait with two heterozygous parents? (2011-2013, 2014-2017) (Answer: B) | 8255 | 42.5% | 53.7% | 11.3% | 26.6% | <0.001 |
| **E** | IK6.3 – Which answer shows a 25% chance of the inheritance of a recessive trait from two heterozygous parents? (2013-2014) (Answer: B) | 1213 | 49.6% | 71.9% | 22.3% | 45.0% | <0.001 |

The first version, **I-K6.0** (Appendix A, Table 1B), was in use for the 2009–2010 school year and was administered to 1,826 students.

- I-K6.0: *If rolling your tongue is a recessive trait, which answer would show a 25% chance of parents passing on the gene?*

The correct answer was chosen by 48.0% of students on the PRE assessment and 69.8% on the POST, an improvement of 21.8% with a p-value of <0.001.

The second version, **I-K6.1** (Appendix A, Table 1C), was in use during the 2010–2011 school year and was administered to a total of 1,242 students.

- I-K6.1: *If rolling your tongue is a dominant trait, which answer would show a 3:1 ratio of parents passing on the trait?*

This version corrected **I-K6.0**’s categorization of tongue-rolling as a recessive trait and made clearer that the answer was looking for the *trait* being passed on, not the *gene*. Despite this, the correct answers dropped to 43.0% on the PRE and 53.7% on the POST, an improvement of 10.7% with a p-value of <0.001.

**I-K6.2** (Appendix A, Table 1D) was the longest-used version of the question, being on the assessments from 2011–2013 and 2014–2017. It was administered to 8,255 students.

- I-K6.2: *Which answer shows the inheritance of a recessive trait with two heterozygous parents?*

This version dropped the “tongue-rolling” aspect completely, as there was little evidence to confirm that it is in fact an autosomal dominant trait and changed the focus from the results of the Punnett square to the genotypes of the parents. However, results remained nearly identical to **I-K6.1**, with 42.5% correct on the PRE and 53.7% correct on the POST, an improvement of 11.2% with a p-value of <0.001.

**I-K6.3** (Appendix A, Table 1E) was only used during the 2013–2014 school year and was administered to 1,213 students, before switching back to **I-K6.2**.

- I-K6.3: *Which answer shows a 25% chance of the inheritance of a recessive trait from two heterozygous parents?*

This version maintained the identification of parental genotypes but reintroduced the “25% chance” aspect from **I-K6.0** to give the students more information to work with towards choosing the correct answer. This yielded results very similar to **I-K6.0**, with a PRE of 49.6% and a POST of 71.9%, an improvement of 22.3% with a p-value of <0.001. Despite these being the best results achieved thus far on this series of questions, the following year the question was switched back to **I-K6.2** due to concerns that the students were merely identifying an answer that contained “25%” rather than applying any actual genetics knowledge.

After publishing the outcomes of nearly 20,000 public school students in Baltimore and Philadelphia [16], the authors determined that additional student knowledge assessments would provide little new data. As a result, the assessments were redesigned to conduct a deeper analysis of students’ progress, leading to a new system for categorizing question versions.

Beginning in the 2017–2018 school year, the updated student assessments focused exclusively on questions assessing the students’ attitudes towards science and did not have any knowledge-based questions at all. The following year program staff realized that while it was not expected to yield substantial changes in knowledge questions, they still provided important information about site quality. Moreover, the team decided to explore ways to modify the programing to further enhance students’ mastery of genetics. In 2019, a question focused on knowledge of Punnett squares was reintroduced. This version of the assessment, which was administered to 3,567 students up through the 2022–2023 school year, included a graphic of a family tree of mice (i.e., not zebrafish) with questions that built upon one another, as shown in Figure 1. By focusing more on the process, rather than on simple regurgitation of vocabulary or pattern identification, staff aimed to gain a clearer picture of the students’ understanding of core genetic concepts.

*Note: As the assessment data from in-person BioEYES students and virtual BioEYES students were compared and found to be very similar in terms of student improvement, as shown in Table 2, no distinction is made between in-person and virtual data in the following analysis.*

The third question on the current version of the assessment, question **I-K3.1** (Appendix A, Table 2), has **(C)** as the correct answer. This was chosen by 47.8% of students on the PRE and 50.6% on the POST, an improvement of 3.8% with a p-value of <0.001.

**Appendix A, Table 2. “Punnett Square” question results on Intermediate (7th/8th grade) BioEYES student assessments, 2019-2023.**

| Knowledge Question | n= | % Correct Pre | % Correct Post | Difference | Percent Change | p-value |
| --- | --- | --- | --- | --- | --- | --- |
| IK3.1 – Stuart grows up and has babies with another mouse with white fur like Amelia. What is the estimated probability of the offspring’s phenotype(s)? (2019-present) (Answer: C) | 3567 | 47.8% | 50.6% | 3.8% | 8.1% | <0.001 |

This data includes the 380 virtual learners from 2020-2021, as well as an unknown number of students from 2021-2023 that had hybrid instruction. For example, for time periods when outside visitors or instructors were still not allowed to enter schools, BioEYES staff dropped off equipment and taught virtually.

The two questions that got the best results by far were **I-K6.0** and **I-K6.3**. The next best results were for questions **I-K6.1** and **I-K6.2**. The worst results were found on the current version of the assessment, question **I-K3.1**.

Questions **I-K6.0** and **I-K6.3** (Appendix A, Table 1) focused more on the students’ ability to identify “25%” of something rather than on their actual genetics knowledge or understanding of what Punnett squares represent. For example, one student remarked, “Something that was confusing was figuring out the Punnett square. It was a little confusing where to put the letters so that they would all match up.” This sentiment was shared by other students as well, suggesting that they were more concerned with the arrangement of the letters than with applying their understanding of what the alleles represented to solve the problem.

Question **I-K6.1** (Appendix A, Table 1), used the terminology of a “3:1 ratio.” For this question, the authors speculate that some students might have struggled with the math skills needed to solve the problem. It is possible that the lower scores were due to students not understanding the concept of “a 3:1 ratio” as well as they understood “25%” of something. Students in Philadelphia and Baltimore learn this content in 6th grade, and take BioEYES in 7th and 8th grade, so in theory they should know it [8–9]. However, math scores were below proficient levels before the pandemic and worsened during the health crisis, which may have also contributed to students’ lack of understanding [9].

Question **I-K6.2** (Appendix A, Table 1) relied primarily on knowing the definition of the word “heterozygous.” While this question requires slightly more knowledge of genetics than the others, it focuses more on understanding definitions rather than on the underlying process.

Question **I-K3.1** (Appendix A, Table 1) required students to follow several steps to demonstrate their understanding of the inheritance process: identifying that a trait is recessive, determining the genotype of an organism exhibiting that recessive trait, and using that genotype to complete a Punnett square, and correctly interpreting its results. This process demanded a much deeper understanding of genetic concepts than previous versions of the knowledge questions, but it was more challenging for the students to grasp. It also required some basic math skills that the students may have been lacking.

**Appendix B. Polygenic Origin of a Trait (slide presentation)**


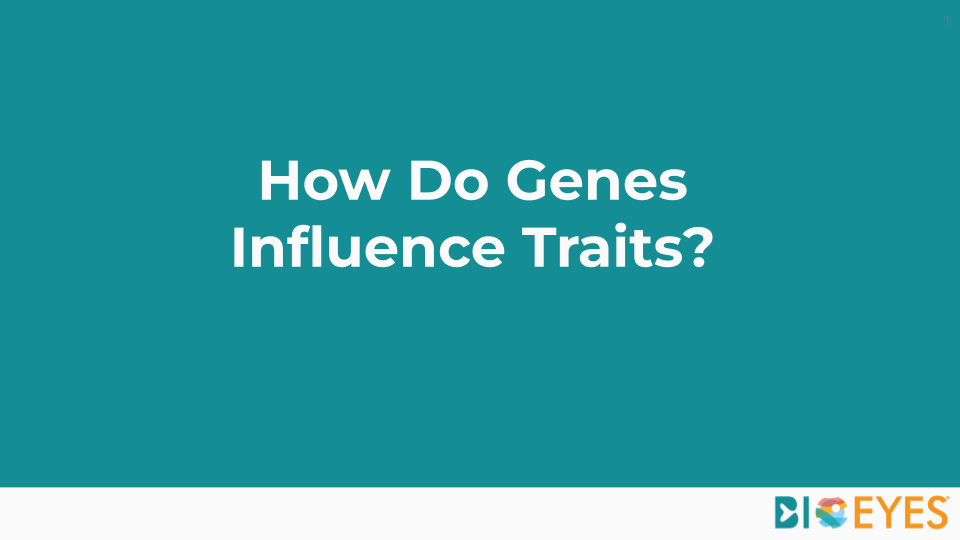

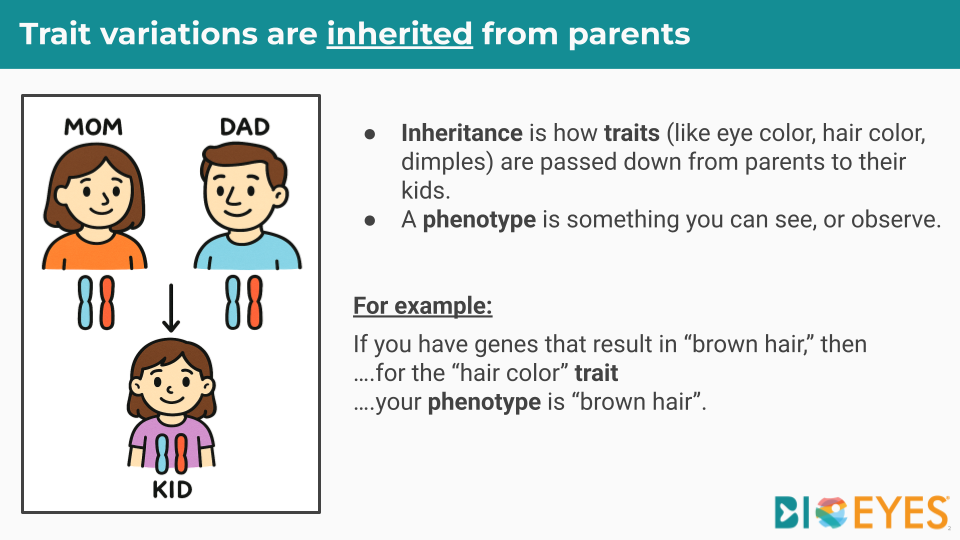

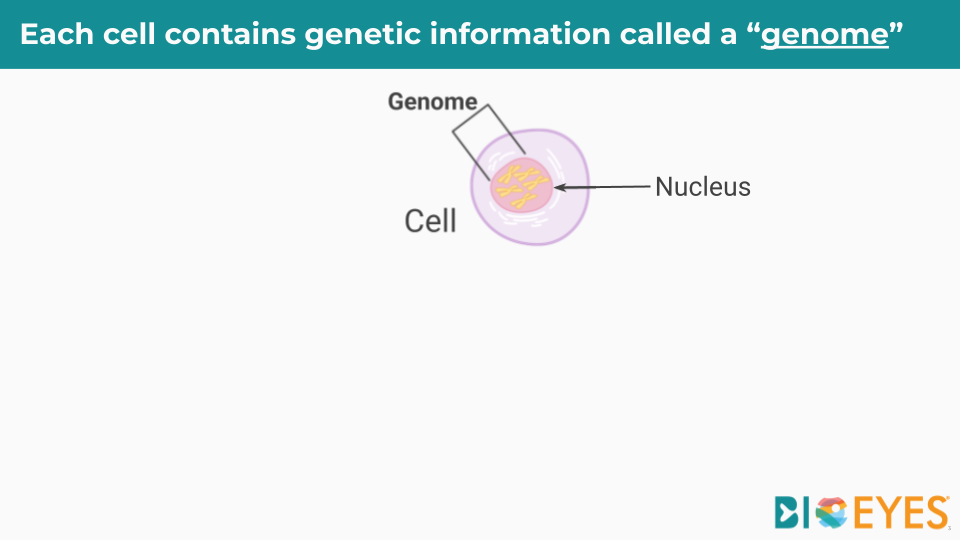

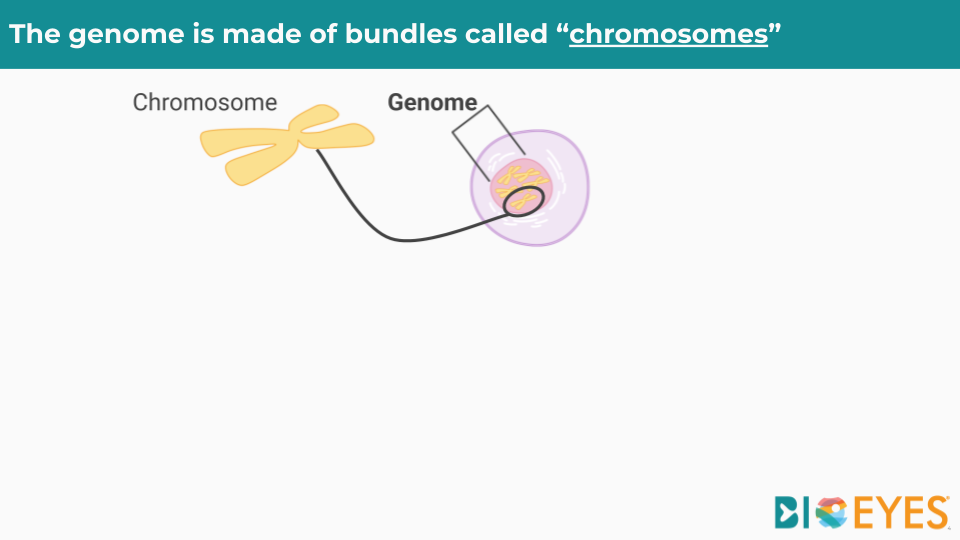

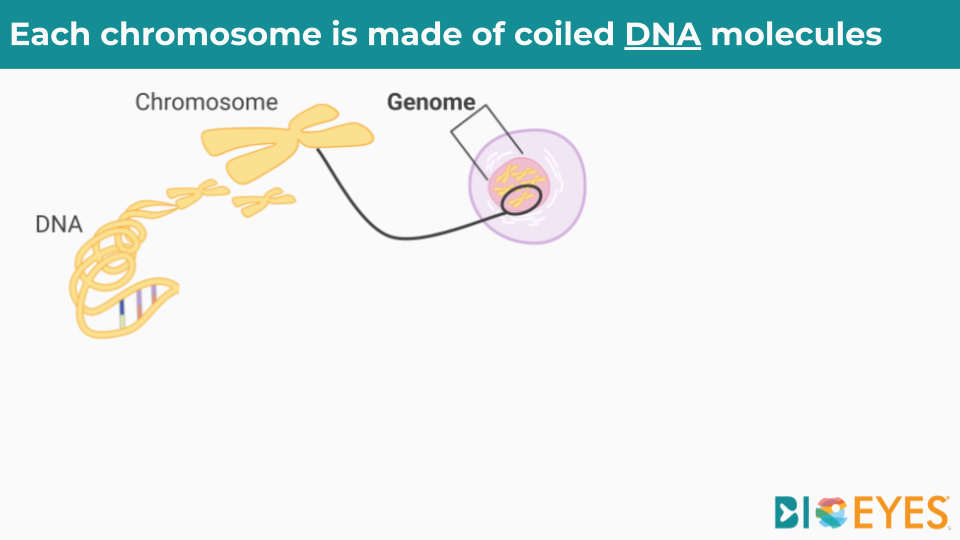

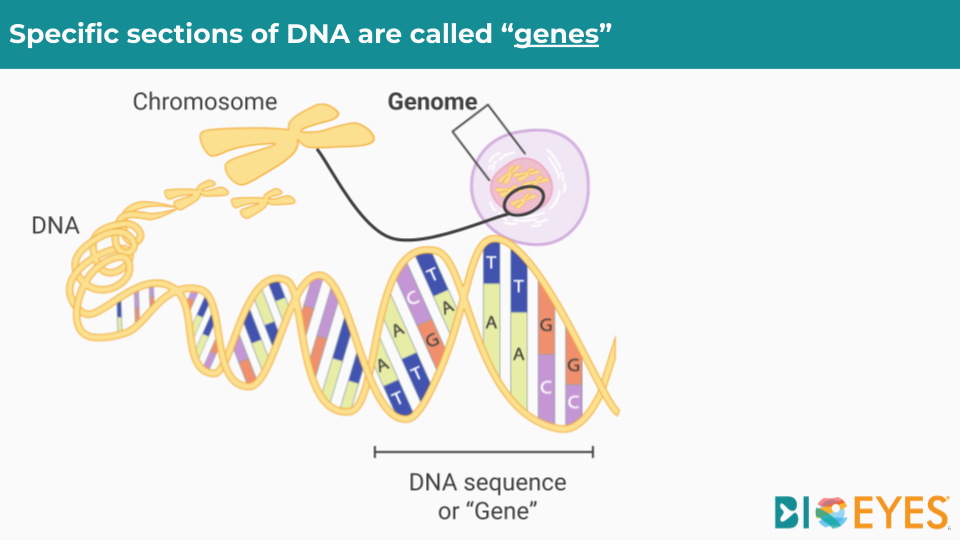

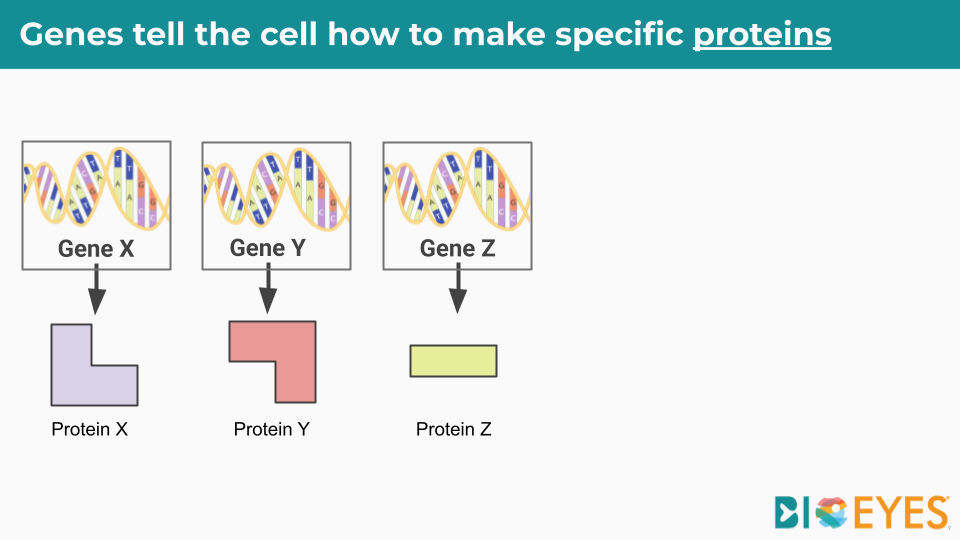

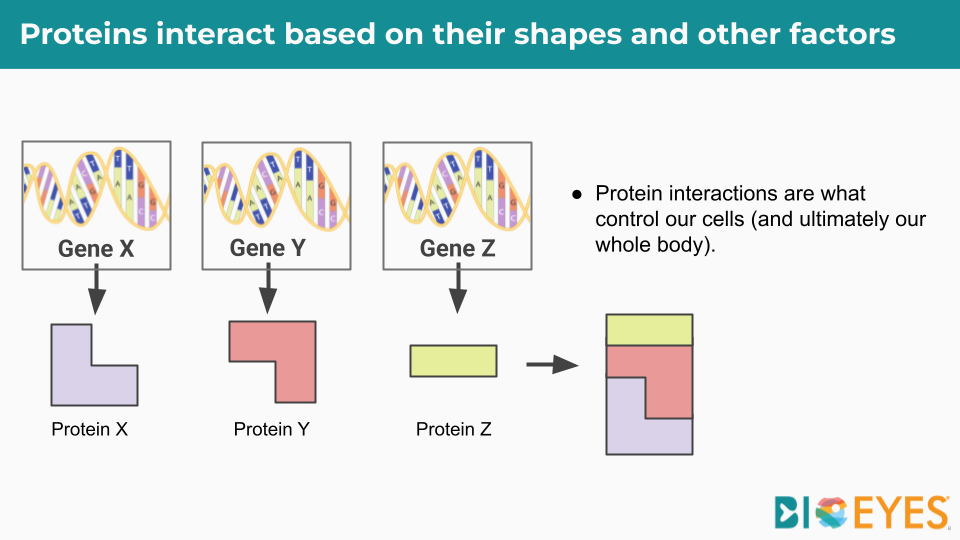

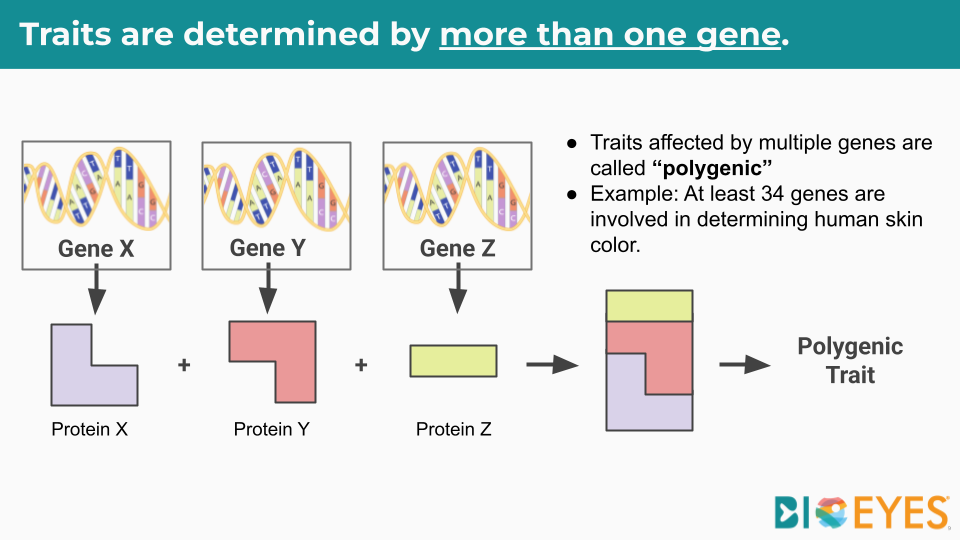

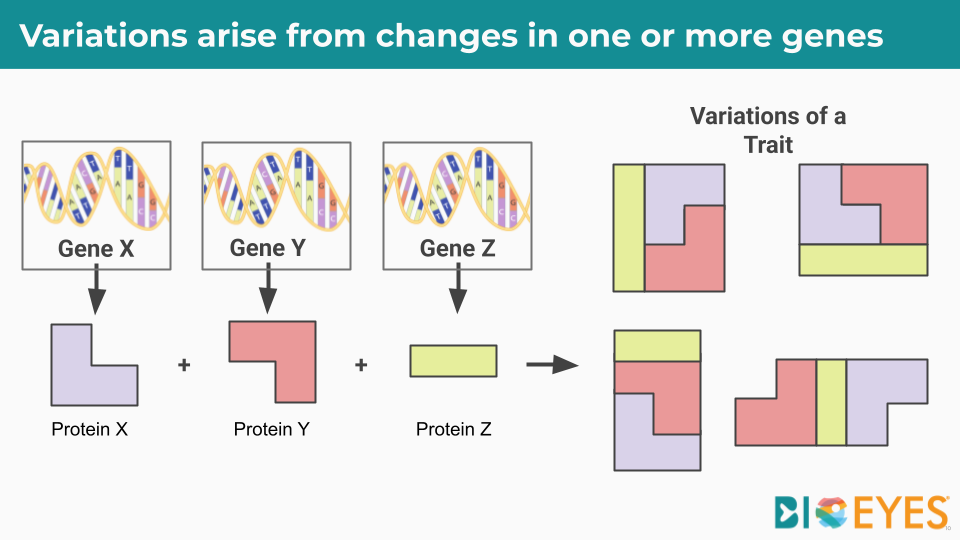

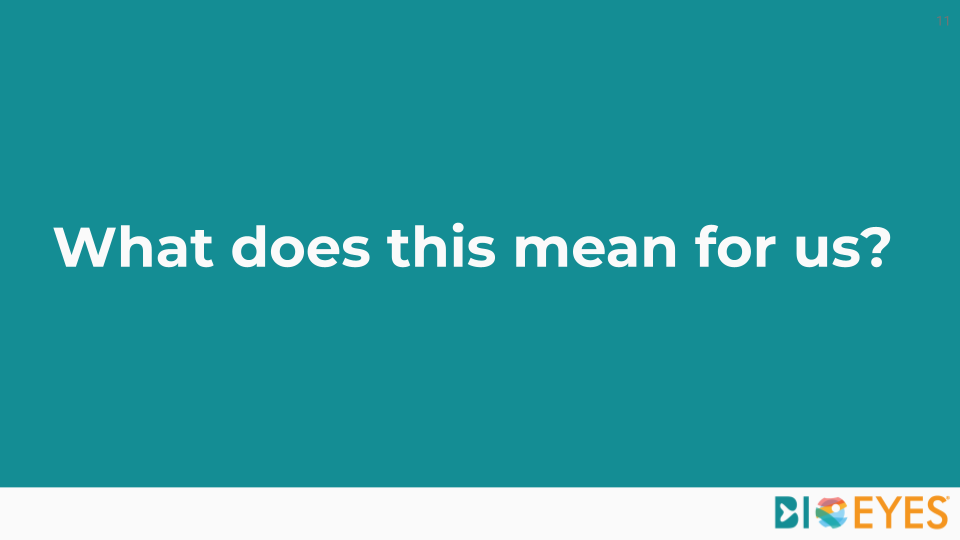

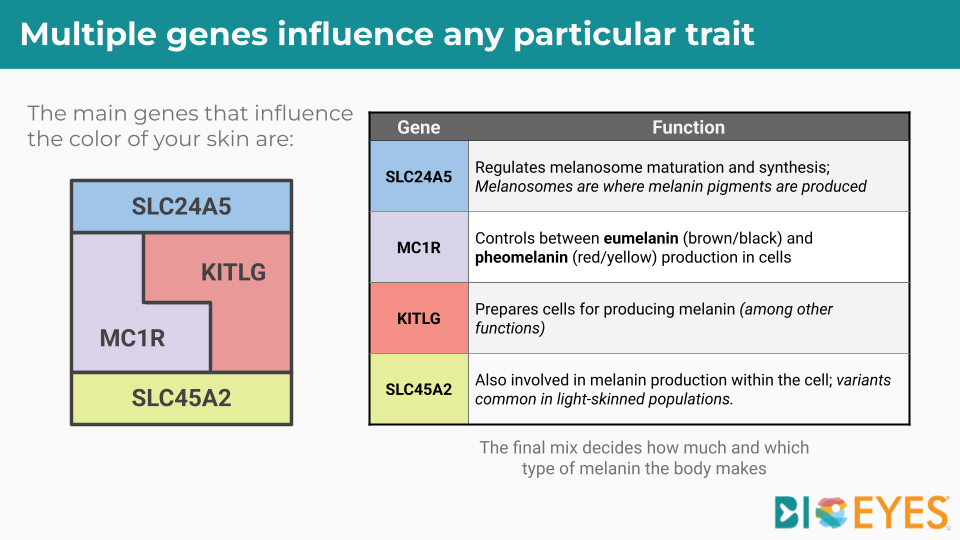

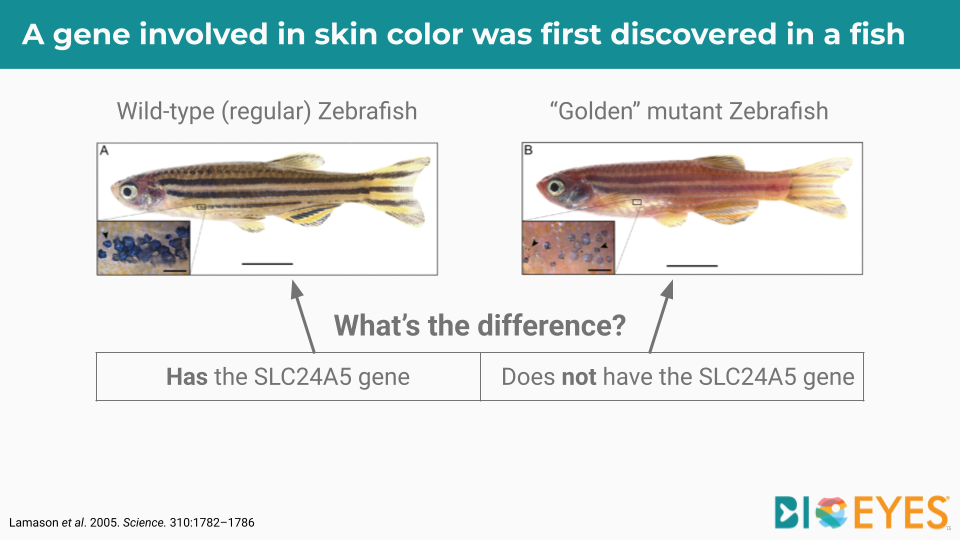

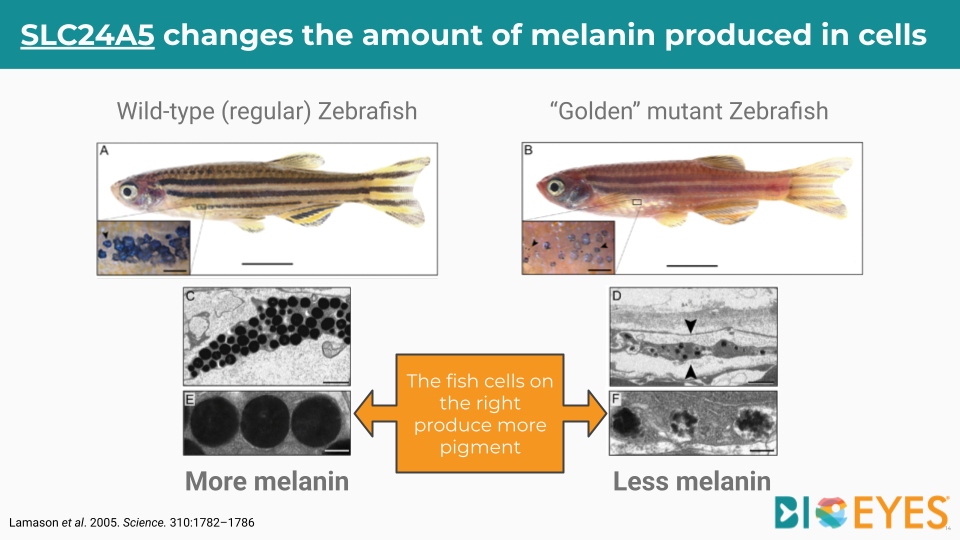

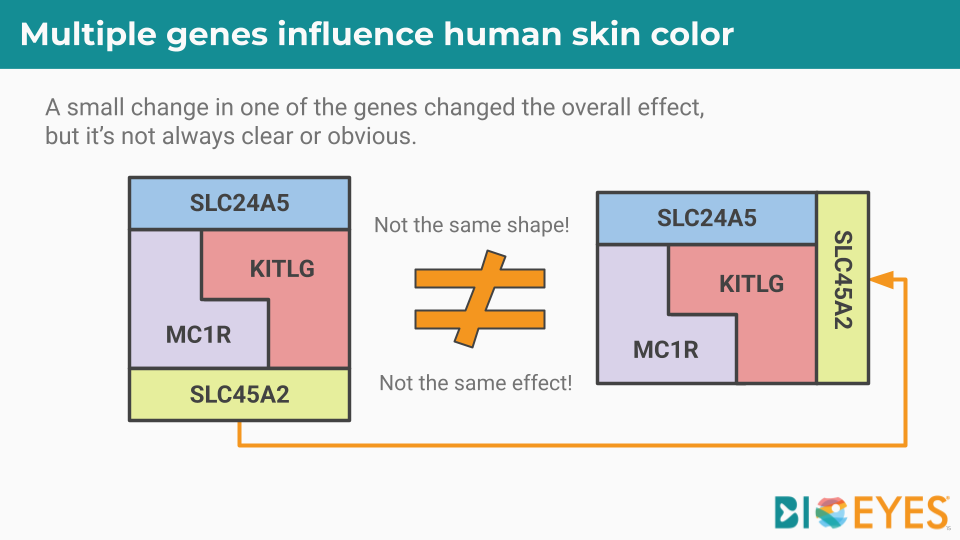

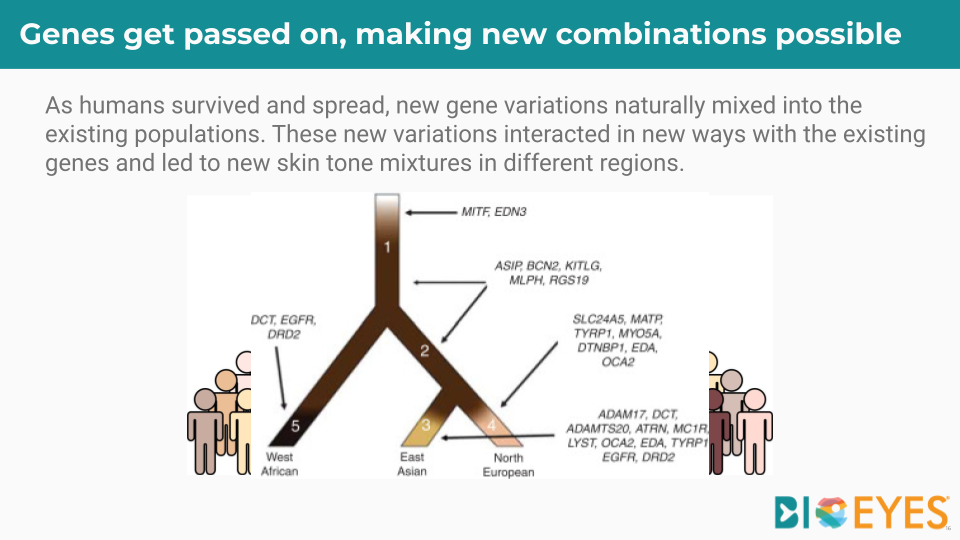

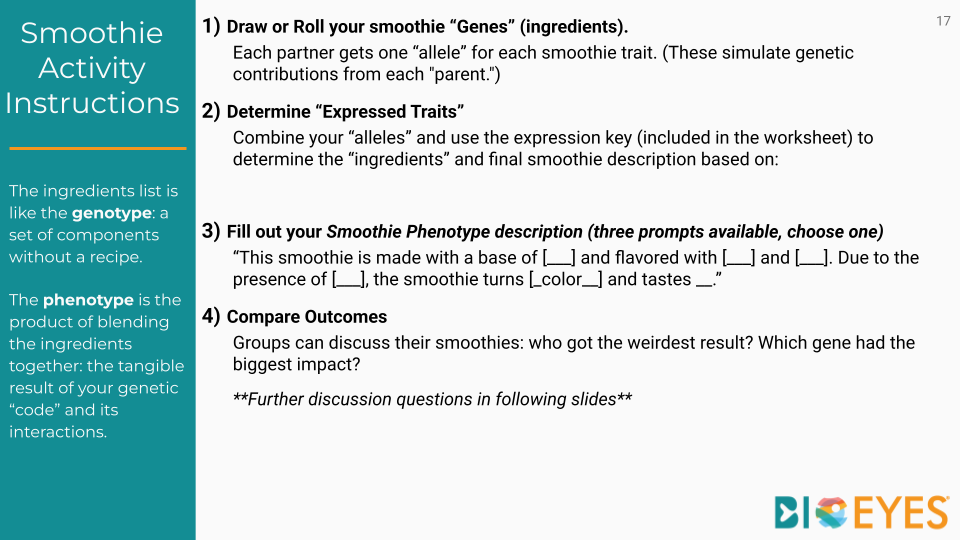

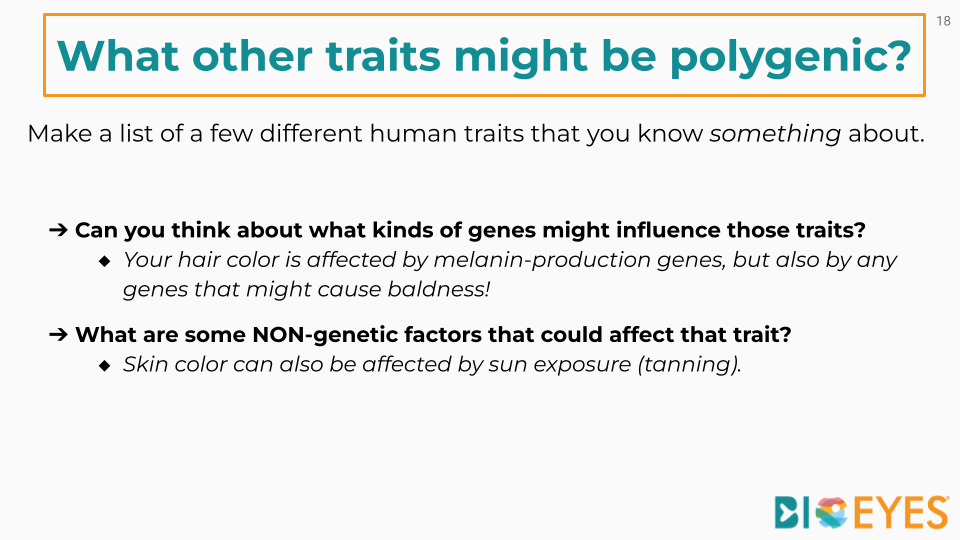

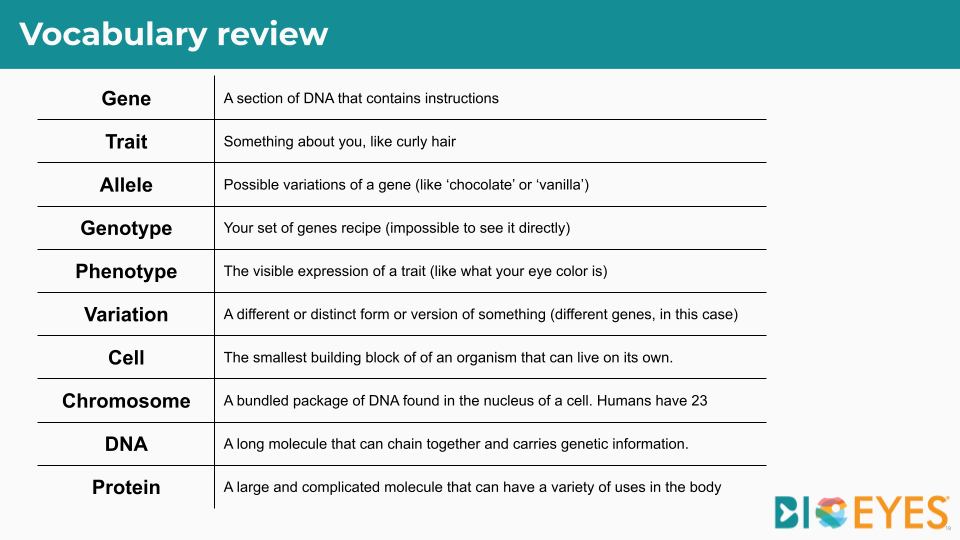

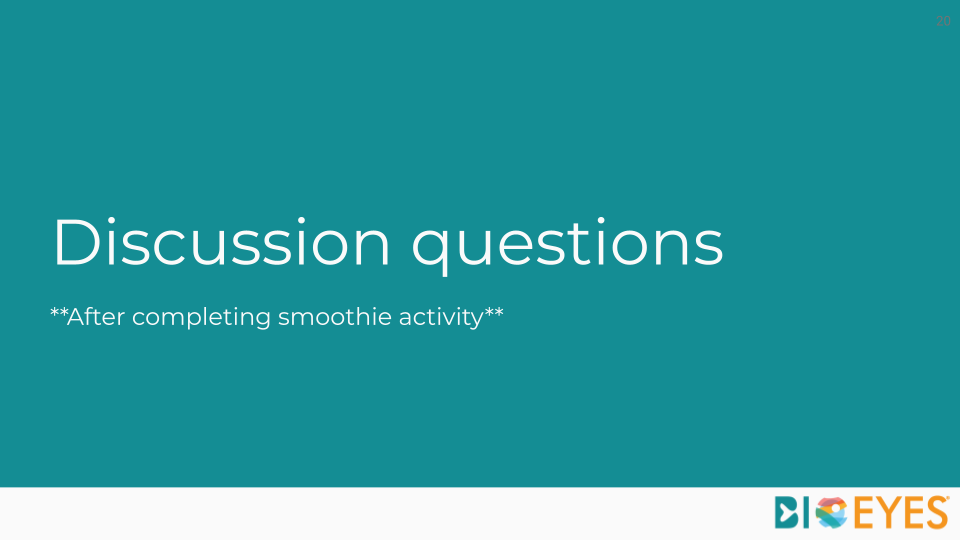

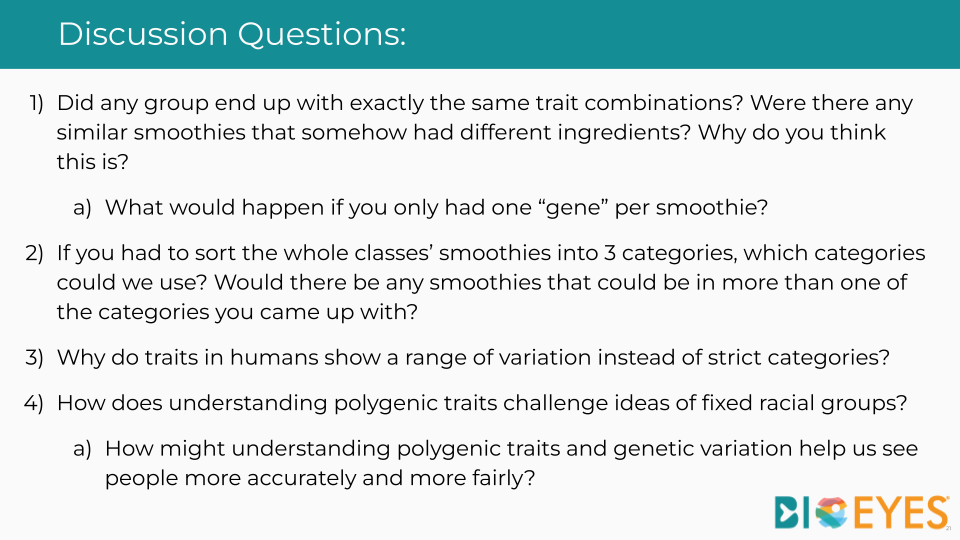


**Appendix C. Smoothie game**

## **Trait Deck:**

## Use this table to print and cut out cards for each category.

If rolling dice, roll once for each category and match to the number in the first column.

| **#** | **BASE** | **FRUIT** | **FRUIT** | **TASTE** | **TEXTURE** |
| --- | --- | --- | --- | --- | --- |
| 1 | Almond Milk | Banana | Pineapple | Honey | Protein  Powder |
| 2 | Coffee | Blueberries | Raspberries | Dates | Ice |
| 3 | Greek Yogurt | Strawberries | Apple | Chocolate | Oats |
| 4 | Orange Juice | Mango | Kiwi | Maple Syrup | Spinach |
| 5 | Milk | Blackberries | Peach | Vanilla  Ice Cream | Coconut  flakes |
| 6 | Water | Papaya | Grapes | Stevia (diet sweetener) | Peanut Butter |

##

##

| **Trait** | **Expression Rule** |
| --- | --- |
| **Base** | Use the thicker liquid (Greek Yogurt > Oat Milk > Almond Milk > OJ > others) |
| **Fruits** | Use both fruits unless duplicated |
| **Taste** | Add both, unless stevia is present. |
| **Effect Gene** | If condition is met (from Effect list), apply corresponding flavor/taste change |
| **Color/ Taste** | Interpret based on effects or additive ingredients (e.g., berries = purple) |

| **EFFECTS** | |
| --- | --- |
| **Milk + Peanut Butter =** Texture is *extra creamy* | **Honey + Maple syrup =** Too sweet! Remove both. |
| **Water + Apple =** flavor is *bland* | **OJ + Raspberries** = taste is *sour* |
| **Stevia =** Cancels other sweetener | **Stevia + Water =** *Diet smoothie* |
| **Ice Cream + other liquid** = Omit liquid and turn into a sundae | **Ice Cream + milk =** *milkshake*  *(even if yogurt is present)* |
| **Blackberries + Raspberries** = removes chocolate or coffee (if present) | **Banana with Spinach =** *Green Smoothie*  **Spinach + any Berry =** *Brown color* |
| **Ice + Peanut butter** = Chunky texture  **Ice + Coconut Flakes** = Chunky texture |  |

## **Student Worksheet Name: _______________ Partner: ____________________**

**Instructions:** Shuffle genes or prepare dice for rolling. Each student draws one “Allele” per category (two per pair for every gene). Record and combine all the results (yours and your partner’s) below.

| **Trait** | **Allele 1** | **Allele 2** |
| --- | --- | --- |
| Base (B) |  |  |
| Fruit (F1) |  |  |
| Fruit (F2) |  |  |
| Taste (S) |  |  |
| Texture (T) |  |  |
| Effect(s) |  | |

###

### **Step 2: Determine Smoothie Traits**

**Instructions:** Use the Expression Key below to determine which alleles are “expressed”.

**Expression Key**

- **Base:** Choose the thicker liquid (Greek Yogurt > Oat Milk > Almond Milk > Orange Juice > Coconut Water > Water)
- **Fruits:** Use ALL fruit alleles. Duplicates make a thicker consistency.
- **Sweetener:** “Stevia” cancels other sweeteners, “Honey” and “Maple syrup” together cancel each other.
- **Texture Add-in:** Combine both add-ins unless an ‘effect’ is triggered.
- **Effect Activated?:** Check if any Effect Gene conditions are met (see Effect Gene list).
- **Final Color/Taste Result:** Imagine effect interactions or combine fruit/sweetness traits to decide (e.g., lots of berries = purple, spinach + banana = green, no fruit = bland.

#

# - Smoothie Description -

- **Base:** ____________________________________________________________
- **Main Fruits:** _____________________________________________________
- **Sweetener:** ______________________________________________________
- **Texture Add-in:** __________________________________________________
- **Effect(s) Activated:** _______________________________________________
- **Final Color/Taste Result:** _________________________________________

###

### **Mad Lib 1: Basic Smoothie**

This smoothie is made with a base of ____________ and blended with _____________ and _____________. It's sweetened with ___________ and includes ______________ for texture.

As a result of the combination, the smoothie turns _____________ and taste is _______________.

### **Mad Lib 2: Unexpected Mutation**

Although we expected a typical smoothie, the combination of _____________ and ______________ triggered an unusual effect: the smoothie turned ______________ or was surprisingly ______________. This was because of the interaction between ___________ and ______________.

### **Mad Lib 3: Polygenic Profile Card**

Alleles from both partners resulted in a smoothie with a base of _____________, fruits like _____________ and _____________, and a texture enhanced by _____________. The sweetness level was influenced by _____________ and modulated by the presence of _____________. This smoothie is a perfect model for polygenic traits—multiple small inputs leading to one complex outcome.

### **Name your Smoothie!** *Based on the taste, texture, and overall appeal of my ingredients.*

Smoothie name: _________________________

# **Appendix D. A lesson plan to teach polygenic gene interaction**

Polygenic Traits Lesson Plan

*8-12th Grade Science Lesson Plan*

*50 minutes - [*[*Lesson Slides*](https://docs.google.com/presentation/d/1GAm4Ow70rOkF2bzCJ5O7s8J0SVWOqwinyen3S8Rqavw/edit?usp=sharing)*]*

**Lesson Objective:**

Students can explain how phenotypic variation is a result of complex interactions between multiple genes (and the environment).

**Essential Question:**

“How do our bodies know what color to make our skin? Why is it different for different people?”

## **Key Vocabulary:**

1. Variation
2. Gene
3. Trait
4. Cell
5. Chromosome
6. Allele
7. DNA
8. Protein
9. Genotype
10. Phenotype

**Materials Needed:**

- Coloring paper and flashlight
- Instructions and prompts for demo
- Some randomizing tool for prompts (dice, a bag to pull numbers from, or students can choose their own)
- Drawing materials

# **Lesson Procedure:**

## **Demonstration Activity (15 minutes):**

1. Students arranged in groups (pairs preferable)
2. Each student receives a worksheet .
3. Students determine the genetic contributions (alleles) for their group based on rolling dice, picking numbers or taking a card (if printed).
4. Students follow instructions from the worksheet to describe the product of the previous steps.

**Extension**: limit resources (dice, materials, etc.) and instruct students to skip any step they do not have materials for, modeling how environmental effects can also impact a trait.

**Genotype–Phenotype Connection:** The ingredients selected represent the genotype: the specific genetic makeup of your smoothie. Each ingredient is like an allele, and together they represent the potential outcomes for your smoothie’s traits. A genotype may include traits that are expressed together, canceled out, or modified by other traits and therefore not visible as part of the final combination.

The taste, texture, and color of your final smoothie represent the phenotype: the observable characteristics that result from the interaction between the genes and their environment. Some phenotypic traits may be subtle (slightly sweeter, thicker) while others are dramatic (bright green from banana + spinach). This mirrors how, in living organisms, the phenotype is a measurable result of genetic instructions interacting with each other and with environmental conditions.

**Lecture (15 minutes):**

- Review concept of inheritance and how it influences physical **traits**:
  - “phenotype” like “**pheno-**menon” and “**type**” (A person with blue eyes has the *‘blue’* **phenotype** for the *‘eye* *color’* **trait**).
- Each of our **cells** contain a string of molecules called DNA that are arranged in a specific pattern.
  - The pattern of DNA molecules works as a code that cells use as instructions to do specific things, similar to how a computer uses electronic code to run different programs or apps (the apps are **made** of code, and there’s also code in the OS that tells the app what to do).
  - DNA gets bundled into bigger structures called chromosomes, so the cell must unravel the DNA to “see” it and can only access certain parts of the DNA at any one time.
- A particular section of DNA that has a distinct *result* is called a “**gene**”.
  - *There are also large sections of DNA that* ***don’t*** *function as genes, similar to digital files that are stored on a device using code similar to that of the apps, but they do not function as programs on their own.*
- There is **variation** within genes. Similar genes might start and end the same, or be in the same/similar position, but the order of the code is changed so they result in slightly different things. *(Like how iPhones and Androids are both cell phones but work differently.)*
- “Genotype” refers to which “version” of the gene we can find in a particular individual’s cells.
- The cell “reads” the genes and uses the information to assemble a protein (which is a type of molecule that can be very complex). These proteins can be used for lots of tasks like making new cells, triggering a particular reaction, or as a step in a chain of reactions.
  - Cells use these proteins to function, but that also depends on the environment! E.g. Average height has increased over the last 200 years, but related genes have not (or not by much), it’s more likely nutrition and disease occurrence that has changed over that time.
- A mutation is when the order of the DNA code in a gene is somehow changed enough to also change the protein it encodes or also alter the effect(s) it produces (like by producing more or less of it).

**Discussion questions:**

1. Did any group end up with the same trait combinations? Why do you think this is?
   1. How would this change if you only had one “Gene” per smoothie?
2. If we had to divide the whole classes’ smoothies into 3 categories, which categories could we use? Would there be any smoothies that would fit more than one of the categories?
3. Why do traits in humans show a range of variation instead of strict categories? (*there are no two people who are exactly the same, even twins have different fingerprints!*)
4. How might understanding polygenic traits and genetic variation help us see people more accurately and more fairly?

### Estimated Total Time: 50 minutes

**Appendix E. Genetics guidelines for teachers**

*For middle and high school teachers*

1. Nature and history of genetics
   - Genetics instruction is a product of the time it was developed
   - People bring their own pre-conceived ideas to the subject that are not always accurate
     1. Historically this has resulted in racial and sexual prejudice
        1. Give examples: Eugenics, White supremacy ideas that still exist today
     2. Therefore, genetics has been used to reinforce pre-existing biases
2. Why Mendel?
   - Background on Mendel, Bateson, and Weldon^1^
   - Mendelian genetics has been the standard instructional tool for over 100 years
   - Mendelian genetics is a simplified model of inheritance that is rare in nature
     1. Instead of one gene producing one trait, it is far more common for multiple genes to interact in myriad ways to produce genetic variation (i.e., mutations)
   - Competition and ego pushed Mendel’s inaccurate model to prominence over other models at the time (Weldon) that more accurately reflect inheritance in nature
3. The polygenic model
   - Have teachers do the Smoothie game
   - Discussion
4. How the environment affects genetic variation
   - Convey an understanding to teachers that the environment affects genetic traits and thus, genes are malleable.
   - Environmental factors such as stress, diet, and exposure to pollution can turn genes on or off.
   - Background on the SLC24A5 gene: Its discovery in zebrafish and humans, how it accounts for about 25%–38% of skin color variation among Europeans and Africans^2^, and 27% between Europeans and those from Middle East, Central Asia, Pakistan, and North India but not South India^3^.
   - Give example of population migration and how a mutation occurred to people that moved north and further away from the equator
     - 1. People that lived closer to the equator where the sun is brighter historically had more melanin but because of natural selection and as populations moved elsewhere, genetic mutations resulted in lighter skin color so people could extract more Vitamin D from the sun’s ultraviolet rays.
     1. Give example of cardiovascular disease: a polygenic disease in that the disease severity is impacted by specific gene variants (e.g. LDL receptor alleles), but also environmental factors such as smoking, exercise, and nutrition.
     2. Discussion
        1. How might social factors affect your genes?
5. Humane Genetics Literacy
   - Background on Brian’s Donovan’s work^4^
   - Activity where we bring up misconceptions in genetics and we discuss them
   - Humans of different “races” are more alike genetically than populations within a single racial category
   - Discussion
6. Summary/Takeaways
   - Genetics instruction is not neutral, and it depends on how it is taught
   - Teaching that polygenic gene interactions often more accurately reflect natural variation than a single gene mendelian model
   - Mendel’s model can be twisted to reinforce racial prejudices. I.e., there is more genetic variation between races, than among them. THIS IS FALSE!
   - Environmental factors like diet, pollutants, stress, or exercise can turn genes on or off; genes are malleable
   - Humane Genetics Literacy: Teaching all the above and minimizing Mendel’s contributions has been shown to reduce racial bias.

^1^ Bapty, H. (2023). Must Introductory Genetics Start with Mendel? Sci & Educ 32, 1677–1708. <https://doi.org/10.1007/s11191-022-00361-z>

^2^ Lamason, R.L., et al. (2005). SLC24A5, a Putative Cation Exchanger, Affects Pigmentation in Zebrafish and Humans. Science 310,1782-1786. DOI:10.1126/science.1116238.

^3^ Basu Mallick C, Iliescu FM, Möls M, Hill S, Tamang R, Chaubey G, et al. The light skin allele of SLC24A5 in South Asians and Europeans shares identity by descent. PLoS Genet. 2013 Nov;9(11):e1003912. doi: 10.1371/journal.pgen.1003912. Epub 2013 Nov 7. PMID: 24244186; PMCID: PMC3820762.

^4^ Donovan, B.M., et al. (2024). Humane genomics education can reduce racism. Science, 383: 6685, 818-822. DOI:10.1126/science.adi7895
